# Activity-Dependent Remodeling of Synaptic Protein Organization Revealed by High Throughput Analysis of STED Nanoscopy Images

**DOI:** 10.3389/fncir.2020.00057

**Published:** 2020-10-15

**Authors:** Theresa Wiesner, Anthony Bilodeau, Renaud Bernatchez, Andréanne Deschênes, Bastian Raulier, Paul De Koninck, Flavie Lavoie-Cardinal

**Affiliations:** ^1^CERVO Brain Research Centre, Québec, QC, Canada; ^2^Department of Biochemistry, Microbiology and Bioinformatics, Université Laval, Québec, QC, Canada; ^3^Department of Psychiatry and Neuroscience, Université Laval, Québec, QC, Canada

**Keywords:** super-resolution microscopy, synaptic plasticity, synaptic proteins, quantitative image analysis, synapse organization, unsupervised machine learning

## Abstract

The organization of proteins in the apposed nanodomains of pre- and postsynaptic compartments is thought to play a pivotal role in synaptic strength and plasticity. As such, the alignment between pre- and postsynaptic proteins may regulate, for example, the rate of presynaptic release or the strength of postsynaptic signaling. However, the analysis of these structures has mainly been restricted to subsets of synapses, providing a limited view of the diversity of synaptic protein cluster remodeling during synaptic plasticity. To characterize changes in the organization of synaptic nanodomains during synaptic plasticity over a large population of synapses, we combined STimulated Emission Depletion (STED) nanoscopy with a Python-based statistical object distance analysis (pySODA), in dissociated cultured hippocampal circuits exposed to treatments driving different forms of synaptic plasticity. The nanoscale organization, characterized in terms of coupling properties, of presynaptic (Bassoon, RIM1/2) and postsynaptic (PSD95, Homer1c) scaffold proteins was differently altered in response to plasticity-inducing stimuli. For the Bassoon - PSD95 pair, treatments driving synaptic potentiation caused an increase in their coupling probability, whereas a stimulus driving synaptic depression had an opposite effect. To enrich the characterization of the synaptic cluster remodeling at the population level, we applied unsupervised machine learning approaches to include selected morphological features into a multidimensional analysis. This combined analysis revealed a large diversity of synaptic protein cluster subtypes exhibiting differential activity-dependent remodeling, yet with common features depending on the expected direction of plasticity. The expanded palette of synaptic features revealed by our unbiased approach should provide a basis to further explore the widely diverse molecular mechanisms of synaptic plasticity.

## 1. Introduction

Learning and memory at the molecular level is characterized by a remodeling of protein organization at synapses. Neurons can have several thousands of synapses, which contain a dense assembly of a wide diversity of proteins (Micheva et al., 2010). The wealth of the synaptic proteome gives rise to supercomplexes of structural and functional proteins that encode synaptic function through multiple signaling cascades (Frank and Grant, 2017). This allows synapses to respond to a rich variety of stimuli and therefore shape circuit activity (Branco and Staras, 2009). Hence, to understand the molecular mechanisms underlying learning and memory at the circuit level, the heterogeneous synaptic population, beyond the individual synapses, needs to be considered.

Fluorescence labeling combined with optical nanoscopy allows different protein species to be discriminated and their organization to be resolved into subsynaptic nanodomains. Groundbreaking studies using STochastic Optical Reconstruction Microscopy (STORM) on dissociated and slice cultures described the highly organized nano-architecture of synaptic proteins (Dani et al., 2010; Nair et al., 2013; Tang et al., 2016). Further studies have described the nanometric arrangement of several proteins at excitatory and inhibitory synapses, ranging from glutamatergic receptors and scaffold proteins to transsynaptic adhesion proteins, and assessed their activity-dependent re-organization (Nair et al., 2013; Broadhead et al., 2016; Glebov et al., 2016, 2017; Tang et al., 2016; Haas et al., 2018; Hruska et al., 2018; Crosby et al., 2019). However, a particular challenge for quantitative assessment of synaptic remodeling with microscopy is that the changes in synaptic strength are heterogeneously distributed across the neuron and are expressed in diverse forms (Edelmann et al., 2017), such that selection of regions of interest (ROIs) can bias the results. Thus, exploiting an analysis framework that can address synaptic remodeling at the population level and discriminate distinct characteristics of synaptic domains should help to further understand the rules that govern synaptic plasticity.

The identification of pre- and postsynaptic nanodomains or protein clusters is generally performed in manually selected, well-identifiable synapses. This is necessarily influenced by the microscope resolution, labeling quality and specificity, and selected morphological criteria, which can vary across studies. A defining criterion for the identification of a functional synapse has been the presence of pre- and postsynaptic proteins in a close vicinity. Characterization of the organization of pre- and postsynaptic proteins can be performed using several metrics, such as their distance to each other (e.g., Nearest Neighbor Distance), correlation of intensity (Pearson coefficient) and degree of overlap (Manders Overlap coefficient) (Dunn et al., 2011). However, these metrics are sensitive to factors such as signal to noise ratio, labeling density, optical resolution, and signal intensity (Lagache et al., 2018). A further challenge is to determine whether the detected protein clusters are distributed in a spatially organized manner or are randomly distributed to each other (Helmuth et al., 2010; Szoboszlay et al., 2017; Lagache et al., 2018). To address these limitations, distance-based methods that statistically infer spatial association (coupling) between sub-cellular structures have been introduced (Helmuth et al., 2010; Lagache et al., 2013, 2018). Statistical Object Distance Analysis (SODA) was recently developed to analyze automatically and quantitatively the spatial association (coupling) between molecules (or protein clusters) in microscopy images (Lagache et al., 2018). In this approach the distance between protein pairs is measured and the enrichment of protein clusters at a given distance is statistically assessed given the null hypothesis of randomly positioned objects. SODA generates maps of spatially associated (coupled) and randomly positioned clusters (uncoupled), providing quantitative measurements of the association level (coupling index) and of the distance (coupling distance) between two associated objects. This approach allows to compute the probability of finding spatially associated protein cluster pairs at a given distance (coupling probability) (Lagache et al., 2018).

To explore the diversity of activity-dependent remodeling of synaptic proteins at the nanoscale over a large population of synapses, we adapted SODA into Python (pySODA). We used this approach to quantify the coupling properties (probability and distance) of synaptic protein clusters in cultured hippocampal neurons exposed to various stimulation conditions and imaged with STimulated Emission Depletion (STED) microscopy. We applied pySODA to analyse the spatial distribution of different pre- and postsynaptic protein pairs and found them to exhibit variable coupling probabilities and coupling distances. We found that stimuli driving synaptic plasticity caused significant changes in coupling properties for distinct protein pairs. For the protein pair Bassoon and PSD95, both chronic inhibition (leading to synaptic scaling) and an acute stimulus (leading to Long Term Potentiation of synaptic transmission [LTP]) caused an increase in their coupling probability, whereas an acute stimulus leading to Long Term Depression of synaptic transmission (LTD) had an opposite effect. To enrich the characterization of the synaptic cluster remodeling at the population level, we applied unsupervised machine learning approaches to include selected morphological features into a multidimensional analysis. This combined analysis revealed a large diversity of synaptic protein cluster subtypes exhibiting differential activity-dependent changes. Mapping of these changes revealed common features depending on the expected direction of plasticity. Our results provide a new framework to investigate the rich diversity of synaptic remodeling processes from a large population of synapses.

## 2. Materials and Methods

### 2.1. Cell Culture and Neuronal Stimulations

Dissociated rat hippocampal neurons were prepared as described previously (Nault and De Koninck, 2009). In brief, before dissection of hippocampi, neonatal rats were sacrificed by decapitation, in accordance with procedures approved by the animal care committee of Université Laval. Thereafter, dissociated cells were plated on PDL-Laminin coated glass coverslips (12 mm) in a 24 well plate at a density of 200 cells/mm^2^. The growth media consisted of Neurobasal and B27 supplement (50:1) and was supplemented with penicillin/streptomycin (50 U per mL; 50 μg per mL) and 0.5 mM L-GlutaMAX (Invitrogen). Fetal bovine serum (2%; Hyclone) was added at the time of plating. After five days, half of the media was replaced by new media without serum and in which Ara-C (5 μM; Sigma-Aldrich) was added to limit non-neuronal cell proliferation. Thereon the cultures were fed twice a week by replacing half of the growth medium with serum- and Ara-C-free medium.

Acute stimuli to neuronal cultures were performed in HEPES buffered solutions at 37°C at 21-22 DIV. The following solutions were used: high Mg^2+^/low Ca^2+^ (in mM: NaCl 98, KCl 5, HEPES 10, CaCl_2_ 0.6, Glucose 10, MgCl_2_ 5), 0Mg^2+^/Gly/Bic (in mM: NaCl 104, KCl 5, HEPES 10, CaCl_2_ 1.2, Glucose 10, MgCl_2_ 0, Glycine 0.2, Bicuculline 0.01), and Glu/Gly (in mM: NaCl 102, KCl 5, HEPES 10, CaCl_2_ 1.2, Glucose 10, MgCl_2_ 1; Glutamate 0.1, Glycine 0.01); Osmolality: 240–250 mOsm/kg, pH: 7.0–7.35. Incubation lasted 10 min for high Mg^2+^/low Ca^2+^ and 0Mg^2+^/Gly/Bic treatments and 2 min for Glu/Gly stimulation. After the treatment, the cells were directly transferred in a 4% paraformaldehyde (PFA) solution for fixation (See Fixation and Immunostaining). To induce synaptic scaling TTX (2 μM final concentration, Sigma-Aldrich) was added 48, 24, or 4 h before fixation.

### 2.2. Fixation and Immunostaining

Cultured hippocampal neurons were fixed at 21-22 DIV for 10 min in freshly prepared 4% PFA solution containing: 4% Sucrose, 100 mM phosphate buffer, 2 mM Na-EGTA. The solution was adjusted to pH 7.4 and used at 37°C. Fixed cells were subsequently washed three times for 5 min with phosphate buffer saline (PBS) supplemented with 100 mM Glycine. Before immunostaining, cells were permeabilized with 0.1% Triton X-100 and blocked with 2% goat serum (GS) for 30 min. Incubation with primary (PAB) and secondary antibody (SAB) was performed in a 0.1% Triton X-100 and 2% GS PBS solution at room temperature (see Table 1). PAB were incubated for 2 h followed by 3 washes in PBS. SAB and Oregon Green 488 Phalloidin (Invitrogen (A12379), dilution 1:50) (Baddeley et al., 2011) were incubated for 1 h and finally washed 3 times in PBS. Coverslips were mounted in Mowiol-DABCO for imaging.

**Table 1:** Primary and secondary antibodies used for STED imaging.

**Primary antibodies**					
**Antibody**	**Company**	**Catalogue no**.	**Dilution**	**Epitope**	**Reference**
Mouse anti-PSD95 (6G6-1C9)	Abcam	MA1-045	1 : 500	Purified recombinant rat PSD-95	Ladépêche et al. (2018)
Mouse anti-Bassoon	Enzo	ADI-VAM-PS003	1 : 500	AA 3569 to 3769 from rat Bassoon	Dani et al. (2010); Tang et al. (2016)
Rabbit anti-Bassoon	Synaptic Systems	141003	1 : 500	AA 3569 to 3769 from rat Bassoon	Dani et al. (2010)
Rabbit anti-Homer1c	Synaptic Systems	160023	1 : 500	AA 152 to 354 from human Homer1b/c	Lagache et al. (2018)
Rabbit anti-RIM1/2	Synaptic Systems	140203	1 : 500	AA 1 to 466 from rat Rim2	Dani et al. (2010); Tang et al. (2016)
Rabbit anti-KCC2	Millipore	07-432	1 : 1000	residues 932-1043 of rat KCC2	Doyon et al. (2011)
**Secondary Antibodies**					
**Antibody**	**Company**	**Catalogue no**.	**Dilution**		**Reference**
GAM STAR 635P	Abberior	2-0002-007-5	1 : 250		Durand et al. (2018)
GAM Alexa 594	Thermofisher	A11005	1 : 100		Durand et al. (2018)
GAR STAR 635P	Abberior	2-0012-007-5	1 : 250		Durand et al. (2018)
GAR Alexa 594	Thermofisher	A11037	1 : 100		Di Biase et al. (2009)

### 2.3. STED Microscopy

STimulated Emission Depletion microscopy (Hell and Wichmann, 1994) was performed on a 4 color Abberior Expert-Line STED system (Abberior Instruments GmbH, Germany), equipped with a 100x 1.4NA oil objective, a motorized stage, and auto-focus unit. Imaging of synaptic proteins labeled with Alexa 594 and STAR 635P was performed with 561 and 640 nm pulsed excitation lasers and a single pulsed 775 nm depletion laser (40 MHz). Fluorescence was detected on two avalanche photodiodes (APD) using a ET685/70 (Chroma, USA) filter for STAR 635P and 615/20 (Semrock, USA) filter for Alexa 594. Phalloidin Oregon Green 488 was excited in confocal mode using a 40 MHz excitation laser at 485 nm and the fluorescence was detected on a third APD with a 525/50 (Semrock, USA) fluorescence filter. Scanning was conducted in a line scan mode with a pixel dwell time of 30μs and pixel size of 15 nm. Line repetitions of 5 and 3 were selected for the STAR 635P and Alexa 594, respectively. Confocal detection pinhole was set around 1 Airy unit. Spectral unmixing was performed using the ImageJ Spectral Unmixing Plugins (Zimmermann et al., 2002; Neher and Neher, 2004; Schindelin et al., 2012). The lateral resolution was approximated by measuring the Full Width at Half Maximum (FWHM) on the fitted line profiles (Lorentzian fit) of 12 isolated protein clusters (STAR 635P : 67.5 nm, SEM 6.7; Alexa 594 nm : 72.6 nm, SEM 8.7).

### 2.4. SODA Analysis

The Statistical Object Distance Analysis (SODA) algorithm was used to analyze the spatial distribution and relations of synaptic protein clusters in STED images (Lagache et al., 2018). Initially developed as a plugin within the Icy image analysis software (De Chaumont et al., 2012), SODA was adapted as a stand-alone Python analysis routine to improve its integration into other high throughput analysis frameworks. SODA requires binary images of segmented protein clusters for each channel (561 and 640 nm) as well as a mask of the region of interest (ROI) comprising the neuronal processes. To generate the ROI mask, we applied a gaussian blur (standard deviation of 10) on the sum of both STED channels and subsequently thresholded the image using 50% of the mean intensity value. Large fields of view (2,000 μm^2^) were acquired and clusters were automatically segmented using wavelet transform decomposition (Olivo-Marin, 2002). The wavelet segmentation parameters (scales 3 and 4) were chosen to discard small low intensity clusters due to non-specific staining and to avoid undesirable separation of clusters. Detected clusters with an area <5 pixels and a width/height <3 pixels were removed. The weighted centroids of the detected clusters were calculated on the raw STED images and only clusters inside the foreground mask were considered for the SODA analysis.

SODA is based on Ripley's *K* function (Ripley, 1976) with an additional boundary correction element *k* (Haase, 1995).

(1) $$\begin{array}{l} K\left(r\right)=\frac{\text{Volume of ROI}}{n_{1}n_{2}}\underset{x,y}{\sum }1\left\{d\left(x,y\right)\leq r\right\}k\left(x,y\right) \end{array}$$

where *n*_*i*_ is the number of objects in the channel *i*. This function is used to count objects that are separated by a distance *d*(*x, y*) shorter than a radius *r*. For SODA, incremental subtractions of the *K* function for a series of distances *r* (corresponding to the pixel size of 15 nm in our experiments) are used in order to count objects from different channels that are within specific distance intervals. This creates the vector

(2) $$\begin{array}{l} G={\left[K\left(r_{i+1}\right)-K\left(r_{i}\right)\right]}_{i=0..N-1} \end{array}$$

where *N* is the number of rings (see Supplementary Figure 1). With a large enough amount of objects (>100, generally reached in STED imaging of synaptic proteins for a large field of view of 2,000 μm^2^), each component *G*_*i*_ of ***G*** is normally distributed with mean μ_*i*_ and standard deviation σ_*i*_, forming vectors **μ** and **σ**. The reduced Ripley's vector

(3) $$\begin{array}{l} G^{0}=\frac{1}{\sigma }A^{-1}\cdot \left[G-\mu \right] \end{array}$$

is used to detect these enriched rings, where ***A*** is a matrix that corrects for the overlap between rings. Only elements of ***G***^0^ that are higher than a universal threshold *T*(*N*) = 2log(*N*) (Donoho et al., 1997) are deemed significant and are conserved; the other elements are set to 0. The coupling probability *P*(*x, y*) for each pair of objects (*x, y*) in different channels can then be calculated with

(4) $$\begin{array}{l} P\left(x,y\right)=\overset{N-1}{\underset{i=0}{\sum }}1\left\{r_{i}<d\left(x,y\right)\leq r_{i+1}\right\}\frac{\sigma_{i}G_{i}^{0}1\left\{G_{i}^{0}>T\left(N\right)\right\}}{G_{i}} \end{array}$$

where 1{} is the Iverson bracket and equals 1 if the inequality inside the brackets is true and 0 otherwise.

In our pySODA workflow, 16 rings with a width of 15 nm were used, for a maximal distance of 240 nm, which is further than the largest distance described between synaptic elements such as Bassoon and Homer1c (Dani et al., 2010).

### 2.5. Multidimensional Analysis

Multidimensional analysis was performed on all acquired 2-color images of PSD95 and Bassoon. Analysis was performed independently for chronic (Naive, 48 h TTX) and acute (high Mg^2+^/low Ca^2+^, 0Mg^2+^/Gly/Bic, Glu/Gly) stimuli. Only coupled clusters were considered. Morphological features were obtained from the detected clusters in the wavelet segmented image consisting in : (1) area, (2) eccentricity, (3) perimeter, (4) major, and (5) minor axis length. These were further combined with the coupling distance and probability that were obtained from the pySODA analysis, resulting in a 7 dimensional feature space. All features were normalized to [0, 1] range using a min-max scaling. A Uniform Manifold Approximation and Projection (UMAP) was used to embed the 7-dimensional feature space onto a 2-dimensional plane (McInnes et al., 2018). UMAP analysis was performed using the provided Python implementation of this dimension reduction technique. We used a number of 25 neighbors to make a compromise between local and broad structure in the input feature space. A minimal distance of 0.05 in the embedded space was used to avoid compressing points together too tightly and allow more sparse local structures.

The embedded feature space was characterized by estimating the density of points using a bivariate kernel density estimation (KDE) of the UMAP for each stimuli. Ten uniformly spaced contour levels were generated for visualization. The local average cluster features (morphological, distance and coupling) was overlaid on the KDE maps to give better insight into each feature's evolution. Hence, it allowed an improved visualization of the remodeling that occurs during stimulation and therefore enabled correlation of feature changes with activity. Local maxima in the KDE map, depicting different subtypes of synapses, were identified and their position was estimated using *peak_local_max* implemented in Scikit-Image (van der Walt et al., 2014). The average features from each local maximum were extracted to create a 7-dimensional vector describing each local maximum, termed synaptic subtype, in the KDE plot.

To group synaptic subtypes with similar features, agglomerative hierarchical grouping (also referred to as hierarchical clustering) was performed using the feature vector corresponding to each local maximum of the KDE plots. The *AgglomerativeClustering* function from Scikit-Learn with euclidean affinity and Ward linkage was used to perform hierarchical grouping (Pedregosa et al., 2011). We calculated the number of groups (synaptic subtypes) that best describe our data based on maximization of the silhouette score, a measure of similarity within a group and dissimilarity between different groups (Rousseeuw, 1987; Zhao et al., 2018).

To investigate the association between the groups identified with hierarchical grouping in the acute and chronic stimulation datasets, we projected all instances (detected protein clusters) of one dataset onto the other dataset and vice versa. We used the Euclidean distance to assign each detected protein cluster to one group. With this approach, we computed the proportion of detected protein clusters of one dataset belonging to each synaptic subtype in the feature space of the other dataset (see **Figures 8B,C**).

### 2.6. Statistical Analysis

Statistical analysis on cumulative frequency curves (CF) and histograms (H) was performed using a randomization test with the null hypothesis being that the different conditions (A, B) belong to the same distribution. The absolute difference between mean values of A and B was calculated for each bin of the CF or H (*D*_gt_ = |μ_*A*_ − μ_*B*_|).

For the randomization test, each value belonging to A and B was randomly reassigned to A' and B', with the sizes of A' and B' being *N*_*A*_ and *N*_*B*_, respectively. The absolute difference between the mean values of A' and B' was determined (*D*_rand_ = |μ_*A′*_ − μ_*B′*_|) and the randomization test was repeated 10,000 times. The obtained distribution was compared with the absolute difference of the mean of A and B (*D*_gt_) to verify the null hypothesis.

When the number of groups was greater than two, the F-statistic was sampled from each group using a resampling method. The F-statistic was calculated from each group (A, B, C, etc.) as a ground truth (*F*_gt_). Each value of the CF or H was randomly re-assigned to new groups (A', B', C', etc.) where group X' has the same size as group X. The F-statistic from each newly formed group (*F*_rand_) was calculated and we repeated this process 10,000 times. We compared *F*_rand_ with *F*_gt_ to confirm the null hypothesis that the groups have the same mean distribution. When the null hypothesis was rejected, i.e. at least one group did not have the same mean distribution, we compared each group in a one-to-one manner using the randomization test described above. In all cases, a confidence level of 0.05 was used to reject the null hypothesis (Supplementary Figures 4, 6, 8, 9, 18, 21).

To calculate the statistical difference between synaptic subtypes, a Chi-square test was used, followed by a *post-hoc* Chi-square test comparing each synaptic subtype with all other subtypes (Supplementary Tables 2, 4, 6, 8).

### 2.7. Localization Error

The weighted centroid of the synaptic clusters was calculated from the intensity image and the synaptic cluster shape using the *weighted_centroid* attributes of the *regionprops* method implemented in Scikit-Image (van der Walt et al., 2014). Hence, a localization error is arising from the uncertainty of the number of counts in the intensity image. The STED setup uses a single photon counting module (Excelitas Technologies, SPCM-AQRH-13) which has an uncertainty on the number of counts of 0.5%. Therefore, using the general error propagation equation one can calculate the uncertainty of localization of the weighted centroid. The calculated localization error is 3 nm.

## 3. Results

### 3.1. Quantitative Assessment of Synaptic Protein Coupling Properties at the Population Level

**Figure 1:**
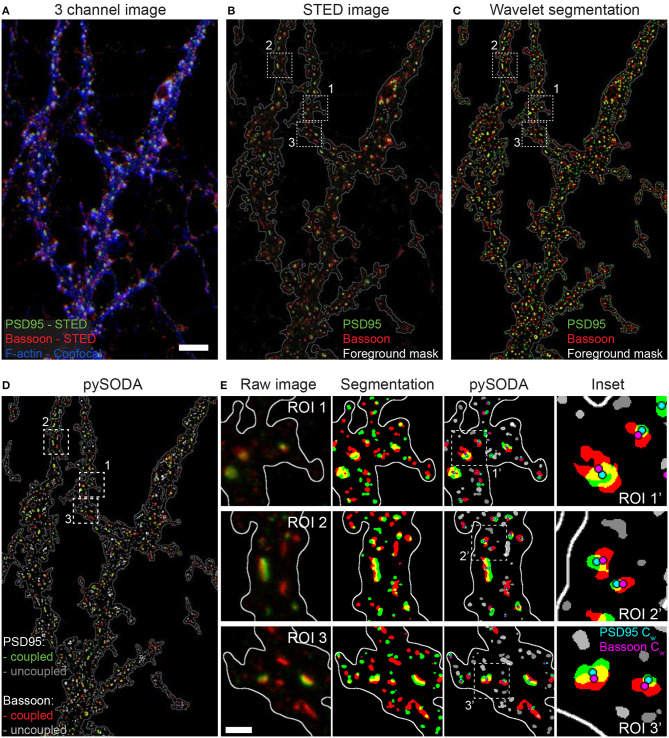
Statistical object distance analysis (SODA) of two-color STED images of the scaffold protein pair PSD95 and Bassoon. **(A)** 3 channel image of a neuron stained with Bassoon-STAR 635P (red), PSD95-Alexa 594 (green) with the corresponding confocal image of Phalloidin-Oregon Green 488 (blue). To better distinguish low intensity clusters combined with the F-actin staining, a log intensity scale was used. **(B)** Raw 2-color STED image (linear intensity scale) showing the region boundaries used for SODA analysis (white contour line). **(C)** Segmented clusters, within the region boundaries, of PSD95 (green) and Bassoon (red) using wavelet transform. **(D)** pySODA analysis of the image shown in **(C)**. Coupled (Bassoon-red, PSD95-green) and uncoupled (Bassoon-light gray, PSD95-dark gray) clusters identified with the pySODA analysis approach. **(E)** Representative regions of interest (ROI) from the image shown in **(D)** (Insets 1-3) showing the raw 2-color STED images *(left)*, the corresponding segmented clusters within the foreground mask *(middle-left)* as well as the coupled and uncoupled clusters identified with pySODA *(middle-right)* and enlarged insets (ROI') from the pySODA map *(right)*. Cyan (PSD95 C_*w*_) and magenta (Bassoon C_*w*_) circles represent the weighted centroids of coupled clusters *(right)*. Scale bars: 5 μm **(A)**, 500 nm **(E)**.

To examine the nanometric distribution of synaptic proteins, we fixed 21-22 DIV primary cultured rat hippocampal neurons and immunostained them for pre- (Bassoon, RIM1/2) and postsynaptic (Homer1c, PSD95) scaffold proteins. We performed two-color STED microscopy of these protein pairs combined with confocal imaging of the F-actin cytoskeleton (stained with Phalloidin-Oregon Green 488) to highlight proximal dendrites with spines (Figure 1A).

To quantify the spatial organization of the protein pairs, we implemented SODA in Python (pySODA). Protein clusters were segmented using wavelet transform, and a foreground mask was generated with a gaussian kernel convolution on the sum of both STED channels (see section Materials and Methods and Figures 1B,C). Using the pySODA framework, the coupling properties between synaptic scaffold protein pairs were calculated (Lagache et al., 2018) (Supplementary Figure 1). The coupling probability was determined for each cluster from the statistical analysis of the measured distances between neighboring clusters of different channels (see section Materials and Methods and Supplementary Figure 1). Coupled clusters were identified when the probability of localizing two synaptic partners at a given distance was greater than the statistical threshold (Lagache et al., 2018) (Figures 1D,E, Supplementary Figure 1, and section Materials and Methods). For each synaptic cluster, pySODA identified neighboring clusters found within concentric rings spaced 15 nm apart (pixel size of the STED images), providing distance-dependent coupling probabilities (Supplementary Figure 1 and section Materials and Methods).

We first assessed the performance of pySODA by measuring the coupling probability of presynaptic Bassoon clusters that were concurrently marked with two distinct secondary antibodies labeled with the fluorophores Alexa 594 and STAR 635P, respectively. As expected for antibodies targeting the same protein, a maximal coupling probability of 0.98 was calculated for clusters found within 15 nm (Supplementary Figures 2A,B). We next evaluated the coupling probability between the presynaptic protein Bassoon and the membrane receptor KCC2 (reported not to be enriched at synapses in basal conditions) (Doyon et al., 2011). As expected, even though the cluster density of KCC2 is high, the calculated coupling probability for Bassoon - KCC2 was very low (0.05, SEM 0.03) and was only above the statistical threshold for clusters spaced by more than 200 nm (Supplementary Figures 2C,D). We also tested whether the increased resolution provided by STED, as compared to confocal microscopy, was necessary for assessing the association between synaptic proteins using the pySODA analysis. We found that the confocal resolution (approximately 235 nm) is not sufficient for such analysis (Supplementary Figure 3).

**Figure 2:**
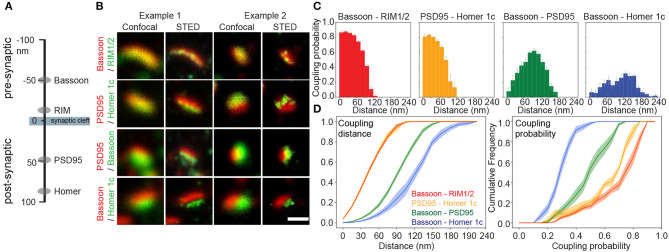
Coupling properties of synaptic scaffold protein pairs at low neuronal activity measured with pySODA. **(A)** Schematic representation of the axial positions of the four synaptic scaffold proteins analyzed in this work. **(B)** Two examples of representative confocal and STED images of synaptic scaffold pairs. **(C)** Coupling probability and distance histograms measured for coupled cluster pairs of Bassoon - RIM1/2 (red, *n* = 16), PSD95 - Homer1c (yellow, *n* = 6), Bassoon - PSD95 (green, *n* = 18), Bassoon - Homer1c (blue, *n* = 17). Each 15 nm bin represents the average coupling probability calculated from the individual images of independent neurons with standard error. **(D)** Cumulative frequency plots of coupling distance *(left)* and coupling probability *(right)* for each synaptic element pair with standard error (shaded area). **(C,D)** *n* = number of neurons from 2 independent cultures. Statistical difference between scaffold protein pairs was assessed using a randomization test (see section Materials and Methods and Supplementary Figure 4). Scale bar 250 nm.

Next, we used pySODA to characterize four different pairs of pre- and postsynaptic scaffold proteins: (1) Bassoon and RIM1/2 (presynaptic), (2) PSD95 and Homer1c (postsynaptic), (3) Bassoon and PSD95 (transsynaptic), and (4) Bassoon and Homer1c (transsynaptic) (Figures 2A,B). Both Bassoon - RIM1/2 and PSD95 - Homer1c protein pairs show maximal coupling probability (CP) between 30 and 45 nm (CP Bassoon - RIM1/2: 0.88, SEM 0.01; CP PSD95 - Homer1c: 0.83, SEM 0.01) (Figure 2C), consistent with the distances measured between these protein pairs with STORM (Dani et al., 2010). Though a similar distribution of coupling distances was calculated for Bassoon - RIM1/2 and PSD95 - Homer1c pairs (Figure 2D, left), the distribution of coupling probabilities for Bassoon - RIM1/2 is shifted to higher levels (Figure 2D, right and Supplementary Figure 4A). This suggests a more organized distribution of Bassoon and RIM1/2 clusters compared to PSD95 and Homer1c.

Since proteins involved in synaptic transmission are thought to be strategically apposed on each side of the cleft (Tang et al., 2016), we examined the coupling properties of two pairs: Bassoon - PSD95 and Bassoon - Homer1c. The highest coupling probability for Bassoon - PSD95 is at a distance of 90–105 nm (CP 0.62, SEM 0.03), while it is between 120 and 135 nm (CP 0.32, SEM 0.03) for Bassoon - Homer1c (Figure 2C), in accordance with distances reported for these proteins (Dani et al., 2010). For Bassoon, the coupling probability was significantly higher with PSD95 than with Homer1c (Figures 2C,D and Supplementary Figure 4F). Indeed >60% of Bassoon - PSD95 couples showed a coupling probability >0.5, while it is only the case for 2% of the Bassoon - Homer1c couples (Figure 2D). These results suggest higher spatial organization between Bassoon and PSD95, as compared to Homer1c. The coupling probability of the transsynaptic partners, as compared to that of the pre- or postsynaptic protein pairs, is necessarily reduced by the fact that a certain proportion of the detected synaptic protein clusters are not part of a functional synapse (i.e., not associated with a pre- or postsynaptic counterpart) (Figure 2C).

Our pySODA framework confirms previous observations regarding the measured distance between synaptic scaffold elements (Dani et al., 2010), while characterizing the coupling properties of the different protein pairs. We thus aimed to use this unbiased approach to analyze synaptic cluster organization at the population level under conditions affecting synaptic plasticity.

### 3.2. Activity-Dependent Stimuli Modulate Coupling Properties of Synaptic Protein Pairs

Recent studies have shown that manipulation of synaptic strength in dissociated cultures can re-organize synaptic scaffold proteins at the nanoscale (Fukata et al., 2013; Tang et al., 2016; Hruska et al., 2018). An increase in synaptic strength can be induced in cultured hippocampal neurons using brief applications of an external solution containing no Mg^2+^, 200 μM glycine and 10 μM bicuculline (0Mg^2+^/Gly/Bic), which drives strong synaptic NMDA receptor activation (Lu et al., 2001; Arnold et al., 2005). We assessed whether such a stimulation alters the coupling properties of synaptic scaffold proteins, by incubating the neurons for 10 min in 0Mg^2+^/Gly/Bic or in an activity-reducing solution containing 5 mM Mg^2+^ and 0.6 mM Ca^2+^ (high Mg^2+^/low Ca^2+^) prior to fixation (see section Materials and Methods) (Lu et al., 2001; Bayer et al., 2006).

**Figure 3:**
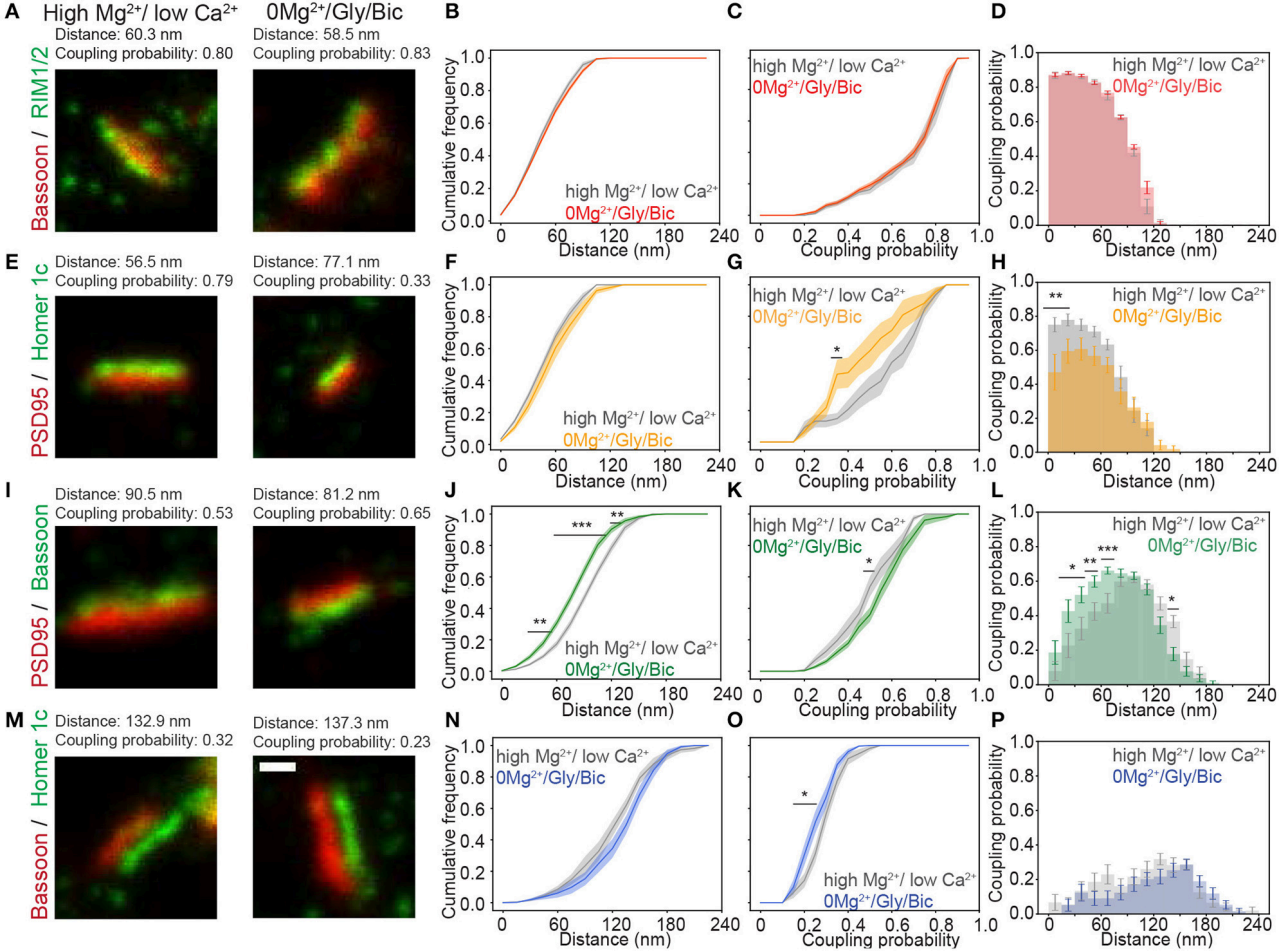
Activity-dependent re-organization of pre- and postsynaptic scaffolding protein pairs measured with the pySODA analysis framework. **(A,E,I,M)** Representative two-color STED images of synaptic protein pairs for the activity reducing high Mg^2+^/low Ca^2+^ condition *(left)* or synaptic stimulation 0Mg^2+^/Gly/Bic *(right)*. Cumulative frequency graphs of the coupling distance **(B,F,J,N)** and of the coupling probability **(C,G,K,O)** measured for coupled protein pairs. **(D,H,L,P)** Histograms of the mean coupling probability per neuron at a given distance. Measurements were performed on two-color STED images of **(A–D)** the presynaptic protein pair Bassoon - RIM1/2 (High Mg^2+^ / low Ca^2+^(gray): *n* = 16 and 0Mg^2+^/Gly/Bic (red): *n* = 21); **(E–H)** the postsynaptic protein pair PSD95 - Homer1c (high Mg^2+^ / low Ca ^2+^(gray): *n* = 9 and 0Mg^2+^/Gly/Bic (orange): *n* = 12); **(I–L)** the transsynaptic pair Bassoon - PSD95 (High Mg^2+^/ low Ca ^2+^(gray): *n* = 18 and 0Mg^2+^/Gly/Bic (green): *n* = 24) and **(M–P)** the transsynaptic pair Bassoon - Homer1c (High Mg^2+^/ low Ca ^2+^(gray): *n* = 17 and 0Mg^2+^/Gly/Bic (blue): *n* = 17). Shown are the means (plain lines) with standard error (shaded area). *n* = number of neurons from 3 independent cultures. Statistical difference was assessed using a randomization test (see section Materials and Methods and Supplementary Figure 6). Exact *p*-values are reported in Supplementary Figure 6, with *p < 0.05, **p < 0.01, and ***p < 0.001. Scale bar 250 nm.

For the Bassoon - RIM1/2 cluster pairs, identified as coupled with pySODA, the mean coupling distance was 59 nm in high Mg^2+^/low Ca^2+^ and remained unchanged upon a 0Mg^2+^/Gly/Bic stimulation (61 nm, Figures 3A,B and Supplementary Figure 5). For both conditions, more than 50% of the coupled Bassoon - RIM1/2 clusters exhibited a coupling probability >0.75, suggesting that the eminent spatial organization of the presynaptic pair is not affected by neuronal activity (Figures 3C,D). By contrast, there was an activity-dependent increase of the mean coupling distance between PSD95 and Homer1c (Figures 3E,F and Supplementary Figure 5), which was associated with a reduced coupling probability (statistically significant for clusters separated by less than 30 nm) (Figures 3G,H and Supplementary Figure 6B). Thus, these two pairs of proteins exhibit different activity-dependent re-organization.

We next assessed whether neuronal activity affects the coupling properties between the transsynaptic protein pairs : (1) Bassoon - PSD95 and (2) Bassoon - Homer1c. For Bassoon and PSD95, increasing neuronal activity yielded a larger population of couples characterized by a smaller coupling distance, combined with an increased coupling probability (Figures 3I–L). Indeed, in the 0Mg^2+^/Gly/Bic condition, a significantly higher coupling probability was calculated for coupling distances between 15 and 75 nm (Figure 3L), while the mean distance between coupled cluster pairs was significantly decreased from 106 to 93 nm (Supplementary Figures 5, 6C). By contrast, synaptic stimulation increased the mean coupling distance between Bassoon and Homer1c and decreased their coupling probability (Figures 3M–P and Supplementary Figure 5). This decrease is consistent with the reduction of the coupling probability measured for the postsynaptic pair Homer1c - PSD95 (Figure 3G). These results suggest that the synaptic distributions of Homer1c with respect to other synaptic scaffolding proteins is regulated by activity. Our results thus show that pySODA is sufficiently sensitive to reveal activity-dependent changes in coupling properties between scaffold protein pairs at the population level.

### 3.3. The Coupling Properties and Morphological Features of Bassoon and PSD95 Are Differently Affected by Long Term Potentiation- or Depression-Inducing Stimuli

In cultured neurons, while the synaptic NMDA receptor stimulation (0Mg^2+^/Gly/Bic) can induce LTP, stimulation of extrasynaptic NMDA receptors can induce LTD (Carroll et al., 1999; Lu et al., 2001). We thus assessed the impact of these LTP- or LTD-inducing stimuli on the coupling properties of the Bassoon - PSD95 pair, by fixing the neuronal cultures immediately after a 10 min 0Mg^2+^/Gly/Bic or 2 min Glu/Gly stimulation.

**Figure 4:**
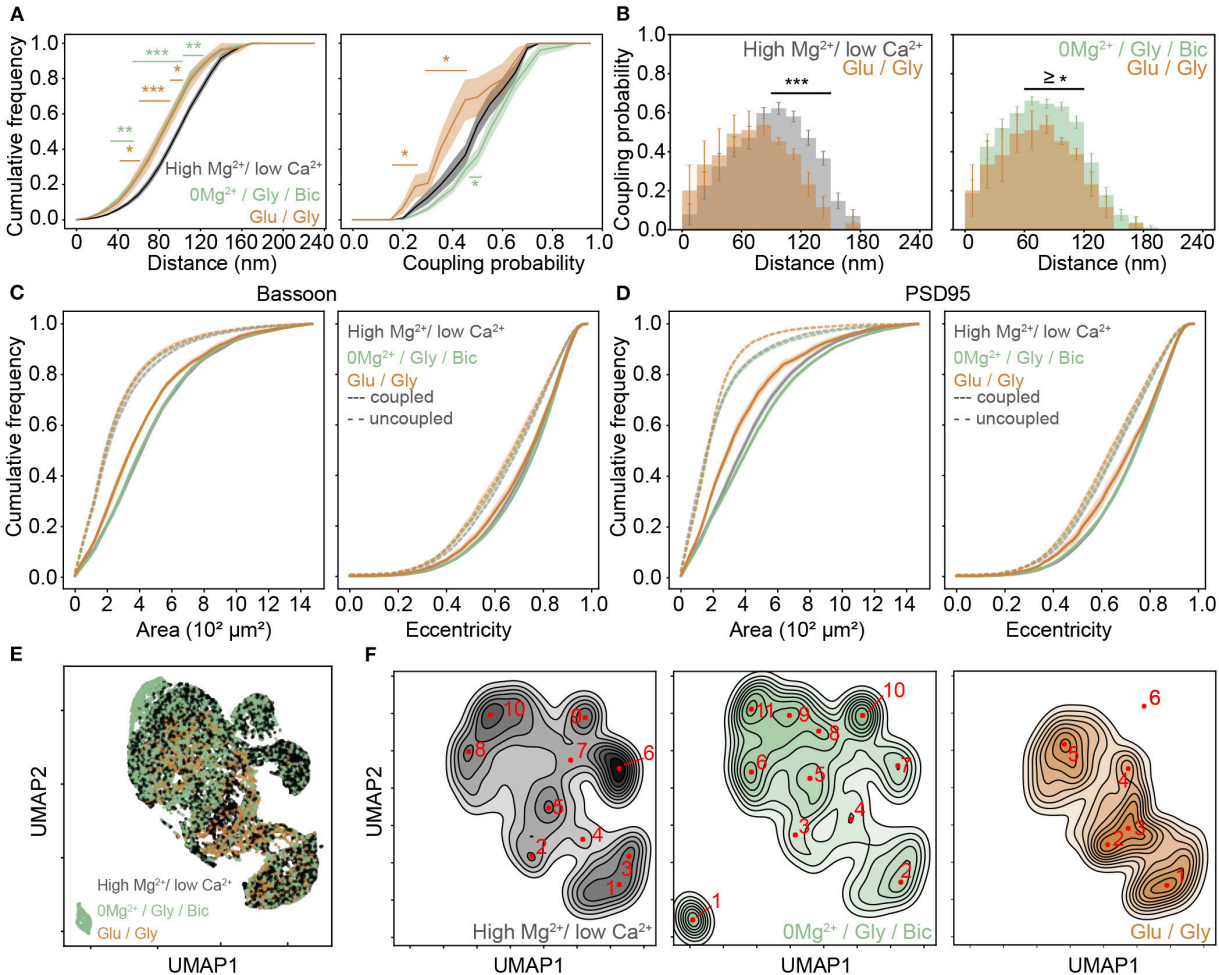
Activity dependent re-organization of Bassoon and PSD95 cluster pairs for different activity promoting stimuli. **(A)** Cumulative frequency curves of the coupling distance *(left)* and probability *(right)* measured for coupled Bassoon and PSD95 clusters in high Mg^2+^/low Ca^2+^ (black, *n* = 18), 0Mg^2+^/Gly/Bic (green, *n* = 24), and Glu/Gly (orange, *n* = 7). *n* = number of neurons from 2 (Glu/Gly) or 3 (high Mg^2+^/low Ca^2+^, 0Mg^2+^/Gly/Bic) independent neuronal cultures. **(B)** Histograms of the mean coupling probability per neuron at a given distance in high Mg^2+^/low Ca^2+^ (gray), Glu/Gly (orange), and 0Mg^2+^/Gly/Bic (green). **(C,D)** Cumulative frequency curves of area *(left)* and eccentricity *(right)* of coupled *(solid line)* and uncoupled (dashed line) Bassoon **(C)** and PSD95 **(D)** protein clusters. (**E**) Scatterplot of the UMAP embedding and **(F)** corresponding kernel density estimation (KDE) maps of coupled Bassoon clusters in high Mg^2+^/low Ca^2+^ (black), 0Mg^2+^/Gly/Bic (green), and Glu/Gly (orange). Local maxima identifying synaptic **(A,C,D)** shown are the means (plain and dashed lines) with standard error (shaded area). Statistical difference was accessed using a randomization test (see section Materials and Methods and Supplementary Figures 8, 9). Exact *p*-values are reported in Supplementary Figures 8, 9 with *p < 0.05, **p < 0.01, and ***p < 0.001.

As shown in the previous section, 0Mg^2+^/Gly/Bic stimulation compared to the high Mg^2+^/low Ca^2+^ condition resulted in an increase of the coupling probability and a reduction of the mean coupling distance of Bassoon and PSD95 (Figures 3J–L and Supplementary Figure 7). In contrast, the Glu/Gly stimulation led to a reduction in both the coupling probability and the mean coupling distance (Figures 4A,B and Supplementary Figures 7,8). These results suggest that while an LTP-inducing stimulus tends to increase the spatial organization between PSD95 and Bassoon, the LTD-inducing stimulus has an opposite effect toward more randomly distributed cluster pairs (Figure 4B and Supplementary Figure 8).

Our results thus far described the activity-dependent changes in coupling properties of synaptic scaffold proteins at the population level. Characterizing additional morphological features of synaptic scaffold protein clusters would be beneficial to appreciate the diversity of the remodeling and to understand further synaptic plasticity (Lagache et al., 2018). We thus added quantitative morphological feature measurements of the Bassoon - PSD95 cluster pairs to the coupling properties analysis. Previous studies (Harris et al., 1992; Matsuzaki et al., 2004) have shown a positive correlation between synaptic strength, spine volume and PSD area. We therefore wondered how the observed changes in coupling probability are reflected in the area and eccentricity of the Bassoon and PSD95 clusters, as well as on their spatial distribution in dendritic shafts and spines. We observed opposing effects of the two stimulation paradigms, with the Glu/Gly treatment decreasing significantly the size of both Bassoon and PSD95 couples (Figures 4C,D and Supplementary Figure 9) and with the 0Mg^2+^/Gly/Bic stimulation increasing of the size of PSD95 clusters (Figure 4D and Figure S9), which was independent from the cluster density (Supplementary Figure 10). In accordance with previous reports (Colledge et al., 2003; Chowdhury and Hell, 2019), we also observed a similar trend in the cluster intensity of coupled PSD95 clusters (Supplementary Figure 11A). We found a higher proportion of coupled Bassoon and PSD95 clusters within spines compared to the dendritic shafts (Supplementary Figure 12).

**Figure 5:**
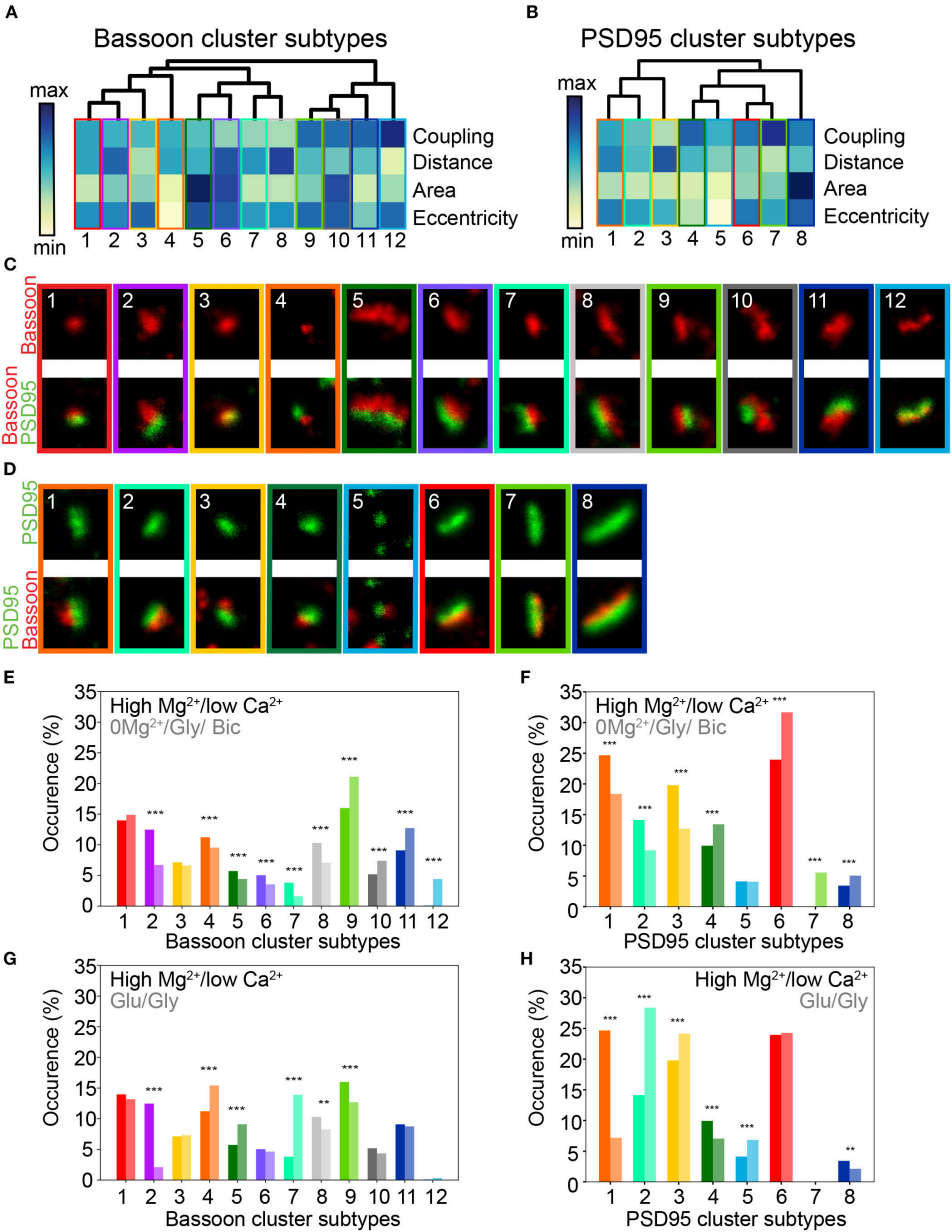
Activity dependent modification of the prevalence of synaptic cluster morphology and organization. **(A,B)** Hierarchical grouping of presynaptic Bassoon **(A)** and postsynaptic PSD95 **(B)** cluster subtypes for 4 selected features (hierarchical grouping including all features are shown in Supplementary Figures 16, 17). Synaptic subtypes were identified from the detected local maxima in the KDE plots generated from the scatterplot of the UMAP embedding of all detected Bassoon and PSD95 clusters (Figure 4, Supplementary Figure 13). Minimum *(yellow)* and maximum *(dark blue)* value of the heatmap for each feature: coupling probability (min: 0, max: 1), distance (min: 0 nm, max: 180 nm), area (min: 0.7·10^−2^ μm^2^, max: 11.8·10^−2^ μm^2^), and eccentricity (min: 0.4, max: 1). **(C,D)** Representative STED images of **(C)** Bassoon and **(D)** PSD95 clusters *(top)* with corresponding two-color STED image *(bottom)*. Proportions of Bassoon **(E,G)** and PSD95 **(F,H)** cluster belonging to each synaptic subtype depending on the neuronal activity state: high Mg^2+^/low Ca^2+^ vs. 0Mg^2+^/Gly/Bic **(E,F)**, high Mg^2+^/low Ca^2+^ vs. Glu/Gly **(G,H)**. Stars represent significant changes in the proportion of synaptic subtypes using Chi-square test. Exact *p*-values are reported in Supplementary Tables 2, 4 with *p < 0.05, **p < 0.01, and ***p < 0.001. Image size: 0.96 μm^2^.

To enrich the characterization of synaptic features in our analysis, we included for each cluster, in addition to the (i) coupling probability and (ii) coupling distance, the (iii) area, (iv) eccentricity, (v) minor axis length, (vi) major axis length, and (vii) perimeter (see section Materials and Methods). To visualize the impact of the different stimulation protocols on these features, we used the dimension reduction technique Uniform Manifold Approximation and Projection (UMAP), which can be used to visualize a high dimensional dataset in a 2-dimensional space (Figure 4E) (McInnes et al., 2018). Local maxima on the Kernel Density Estimate plots (KDE) generated from the scatterplot of the UMAP embedding for each stimulation paradigm were used to identify the major categories of Bassoon and PSD95 clusters (synaptic subtypes) for each stimulation condition (Figure 4F and Supplementary Figure 13). This yielded a wide range of Bassoon and PSD95 cluster subtypes exhibiting different morphological features and coupling properties (Supplementary Figures 14, 15). To identify the synaptic subtypes that are most prominent across the stimulation conditions, we used agglomerative hierarchical grouping (also known as hierarchical clustering) (Figures 5A,B, Supplementary Figures 16, 17, and section Materials and Methods). This approach allows to group observations in a way that the similarity within a group (in our case synaptic protein pairs belonging to one synaptic subtype) is maximized and that the dissimilarity between groups (synaptic subtypes) is also maximized. We relied on the maximization of the silhouette score (Rousseeuw, 1987), a measure of similarity within groups, to determine the number of synaptic cluster subtypes that best describe the properties of the detected protein clusters at the population level and across stimulation conditions (Supplementary Figures 16, 17, and section Materials and Methods). With this approach we identified 12 main groups, which we refer to as synaptic subtypes, for Bassoon and 8 for PSD95 protein clusters (Figures 5A–D). We next measured the euclidean distance between each detected protein cluster and the synaptic subtypes in the multidimensional feature space (comprising morphological features and coupling properties). This allowed to assign each detected cluster to one main subtype and to quantify the prevalence of the synaptic subtypes for each stimulation condition (Figures 5E–H and Supplementary Tables 1, 3).

This analysis revealed that the coupling properties and morphological features of coupled Bassoon - PSD95 pairs are not directly correlated. For example, similar coupling probabilities (e.g., *ST*_*Bassoon*_1-4, CP 0.48–0.55) were associated with a large range of mean coupling distances (53–100 nm) and cluster eccentricities (0.41–0.83), (Supplementary Tables 1, 3). Indeed, the population of coupled Bassoon and PSD95 clusters can be described by subtypes exhibiting a large diversity of complementary coupling and morphological features (Figures 5A–D).

Using this approach we identified three levels of coupling for the detected synaptic subtypes : (1) weak coupling (CP < 0.5), (2) moderate coupling (0.5 > CP < 0.7), and (3) strong coupling (CP > 0.7) (Figures 5A,B and Supplementary Tables 1, 3). As expected from the pySODA analysis, we observed for both Bassoon and PSD95 clusters a significant increase in the proportion of strongly coupled subtypes following the LTP-inducing stimulus 0Mg^2+^/Gly/Bic (*ST*_*Bassoon*_9-12, *ST*_*PSD*95_4,7, Figures 5E,F and Supplementary Tables 1–4). In contrast, the LTD-inducing stimulus significantly increased the prevalence of weakly coupled clusters (*ST*_*Bassoon*_3,5-8, *ST*_*PSD*95_2-3, Figures 5G,H and Supplementary Tables 1–4). When considering morphological features such as area and eccentricity, the 0Mg^2+^/Gly/Bic and Glu/Gly stimulations had an opposite effect on the proportion of small and round subtypes (*ST*_*Bassoon*_4, *ST*_*PSD*95_4-5) that could not be explained solely by the analysis of their coupling properties (Figures 5A,B,E,H). Additionally, we identified for each protein one subtype of small, eccentric and highly coupled clusters (*ST*_*Bassoon*_12, *ST*_*PSD*95_7) that are solely detected following the LTP paradigm (Figures 5E,F and Supplementary Tables 1, 3).

This multidimensional analysis of morphological and coupling properties confirms that both stimuli have an opposite effect on the prevalence of certain synaptic subtypes, and it underlines the diversity in the characteristics and activity-dependent remodeling of the cluster subtypes forming functional synapses. The complementary information on the morphological and coupling properties of the cluster subtypes reveals a more complete description of synaptic plasticity.

### 3.4. Chronic Inhibition of Neuronal Activity Influences the Morphological and Coupling Properties of Bassoon and PSD95

The chronic inhibition of action potentials with the sodium channel blocker Tetrodotoxin (TTX) leads to the strengthening of excitatory synapses, referred to as synaptic scaling (Turrigiano et al., 1998), which is mediated by the insertion of postsynaptic glutamate receptors (Watt et al., 2000) and the remodeling of pre- and postsynaptic scaffold proteins (Sun and Turrigiano, 2011; Glebov et al., 2016). We therefore hypothesized that a prolonged TTX treatment in cultured neurons would lead to an increase in Bassoon and PSD95 coupling probability.

**Figure 6:**
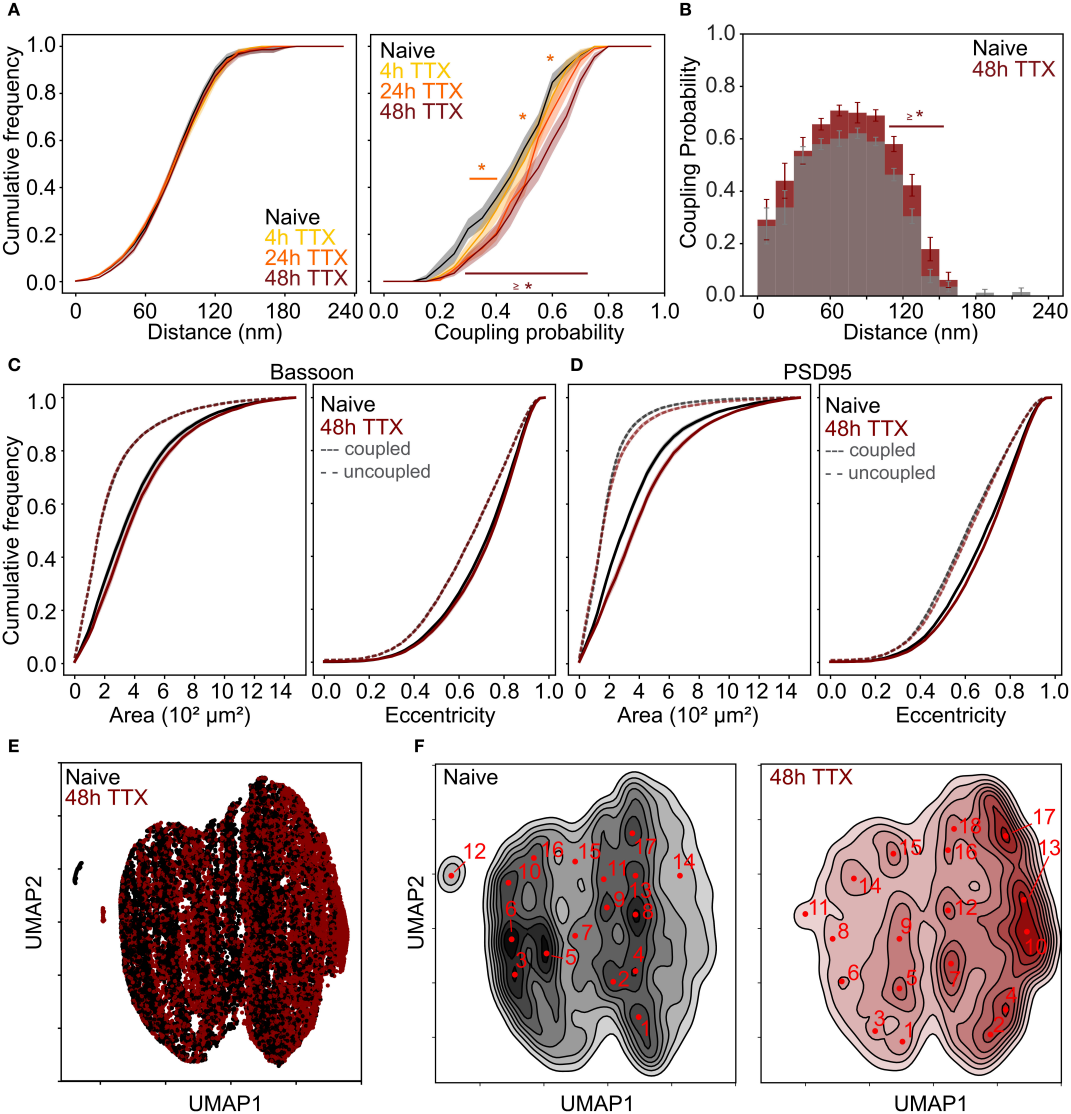
Chronic TTX treatment induced re-organization of the synaptic scaffold protein pair Bassoon - PSD95. **(A)** Cumulative frequency curves of the coupling distance *(left)* and probability *(right)* measured for coupled Bassoon and PSD95 clusters for naive (gray, *n* = 35), 4 h TTX (light orange, *n* = 20), 24 h TTX (dark orange, *n* = 23) and 48 h TTX (red, *n* = 33). **(B)** Histogram of the mean coupling probability per neuron at a given distance for naive (gray) and 48 hours TTX (red). **(C,D)** Cumulative frequency curves of the area *(left)* and eccentricity *(right)* of coupled *(solid line)* and uncoupled *(dashed line)* Bassoon **(C)** and PSD95 **(D)** protein clusters. **(E)** Scatterplot of the UMAP embedding and **(F)** corresponding KDE plots of coupled Bassoon clusters for naive (black) and 48 h TTX (red). Subtypes of synaptic clusters are indicated on the KDE maps (numbers in red) referring to local maxima (see section Materials and Methods). **(A,C,D)** Shown are the means with standard error (shaded area). Statistical difference was assessed using a randomization test (see section Materials and Methods, Supplementary Figures 18, 21). *n* = number of neurons from 2 independent neuronal cultures.

We incubated 21-22 DIV neuronal cultures with TTX for 4, 24, and 48 h prior to fixation. The duration of TTX treatment correlated with increased coupling probability between Bassoon and PSD95, but had no effect on the mean coupling distance (Figures 6A,B and Supplementary Figures 18, 19). The modulation of the coupling probability was also observed when comparing the LTP and LTD conditions to the naive condition (Supplementary Figure 20). The TTX treatment led to a significant increase in the size of the coupled PSD95 (Figures 6C,D and Supplementary Figure 21), the cluster density, number of coupled clusters, and cluster intensity (Supplementary Figures 11B, 22).

**Figure 7:**
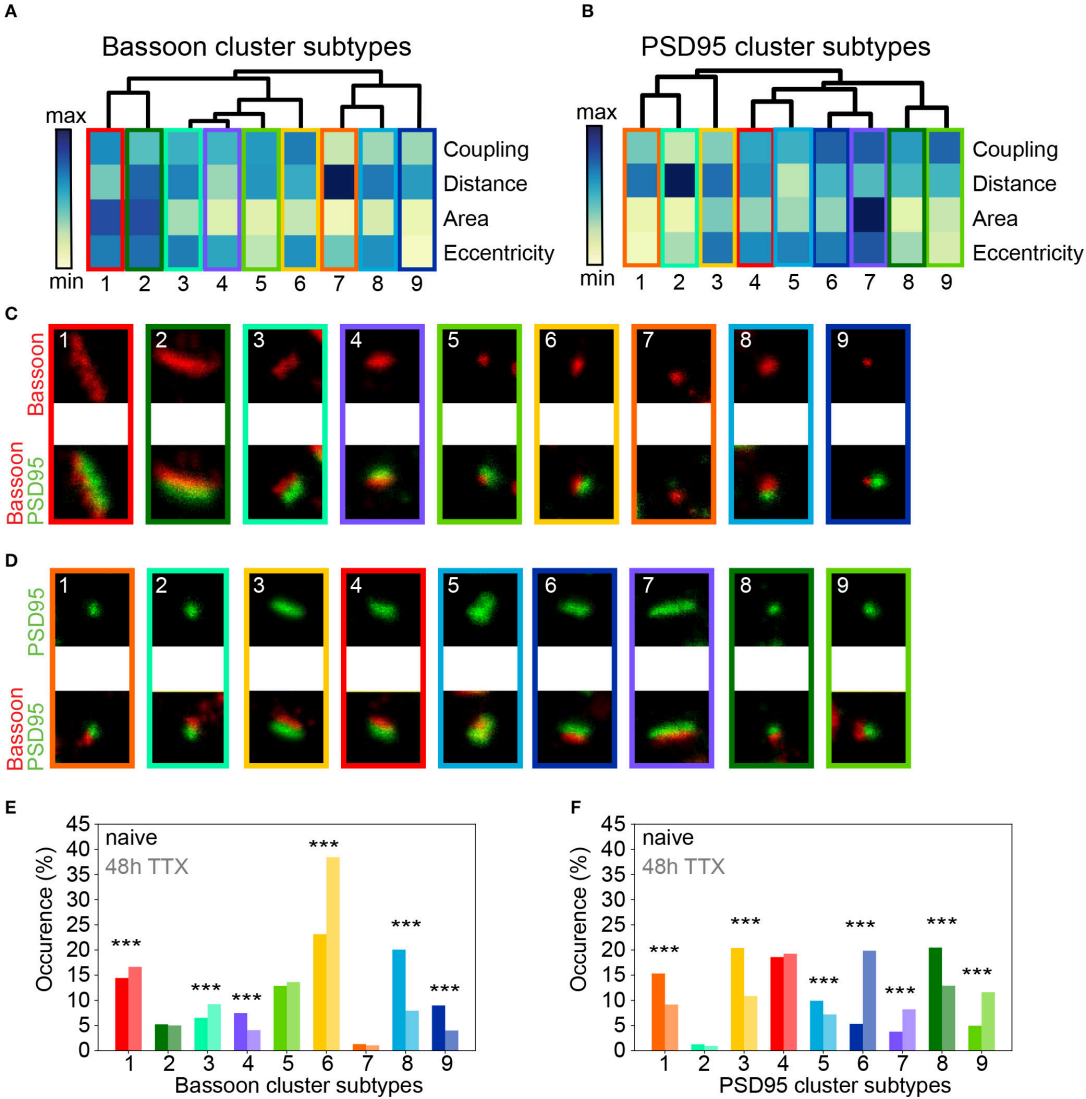
Chronic TTX treatment modifies the prevalence of synaptic cluster morphology and organization. **(A,B)** Hierarchical grouping of presynaptic Bassoon **(A)** or postsynaptic PSD95 **(B)** synaptic subtypes for 4 selected features (hierarchical grouping including all features are shown in Supplementary Figures 26, 27). Synaptic subtypes were identified from the detected local maxima in the KDE plots generated from the scatterplot of the UMAP embedding of all detected Bassoon and PSD95 clusters (Figure 6 and Supplementary Figure 23). Minimum *(yellow)* and maximum *(dark blue)* value of the heatmap for each feature: coupling probability (min: 0, max: 1), distance (min: 0 nm, max: 180 nm), area (min: 0.7·10^−2^ μm^2^, max: 11.8·10^−2^ μm^2^), and eccentricity (min: 0.4, max: 1). **(C,D)** Representative STED images of **(C)** Bassoon and **(D)** PSD95 clusters *(top)* with corresponding two-color STED image *(bottom)*. **(E,F)** Proportions of Bassoon **(E)** and PSD95 **(F)** clusters belonging to each synaptic subtype depending on the neuronal activity state: naive vs. 48 h TTX. Stars represent significant activity-dependent changes in the proportion of each synaptic type using a Chi-square test. Exact *p*-values are reported in Supplementary Tables 6, 8 with *p < 0.05, **p < 0.01, and ***p < 0.001. Image size: 0.96 μm^2^.

We applied again the UMAP-based analysis to characterize the diverse features of the Bassoon - PSD95 synaptic clusters, following chronic TTX treatment (Figures 6E,F and Supplementary Figure 23). Hierarchical grouping identified 9 groups that we refer to as the main synaptic subtypes for each protein (Figures 7A–D and Supplementary Figures 24–27).

Consistent with the results obtained with pySODA (Figures 6A,B), chronic TTX significantly promoted strongly coupled synaptic subtypes (*ST*_*Bassoon*_1,6; *ST*_*PSD*95_6,7,9; CP > 0.6), while the prevalence of weakly coupled subtypes was reduced (*ST*_*Bassoon*_7-9; *ST*_*PSD*95_1-3; CP < 0.4) (Figures 7E,F and Supplementary Tables 5–8). Our results also indicate that compared to basal condition, the 48h TTX treatment strongly reduced the proportion of small synaptic subtypes (area < 0.03 μm^2^) from 49 to 30% (*ST*_*Bassoon*_4,5,8,9) for Bassoon and for PSD95 from 37 to 23% (*ST*_*PSD*95_1,8), (Figures 7E,F and Supplementary Tables 5–8). Interestingly, while the PSD95 cluster population in this homeostatic plasticity paradigm is best represented by similar percentages (10–20%) of the main synaptic subtypes (Figure 7F), a large proportion of Bassoon clusters (38%) belongs to a small, eccentric, and strongly coupled subtype (*ST*_*Bassoon*_6, Figure 7E).

Thus, the pySODA approach combined with multidimensional analysis of morphological and coupling properties revealed that the organization of functional synapses at the nanoscale is modulated by chronic inhibition of action potentials.

### 3.5. Mapping of Synaptic Subtypes of PSD95/Bassoon Reveals Common Features Between Different Forms of Plasticity Induction

This diverse array of synaptic subtypes revealed from hierarchical grouping is the product of non-biased approaches which had no pre-conceived notion of what features of synaptic plasticity should emerge following different stimuli. Hence, the number of groups resulting from this data-driven unsupervised grouping approach intrinsically varied with the number of instances supporting the model and the distribution of these instances in the representation space.

**Figure 8:**
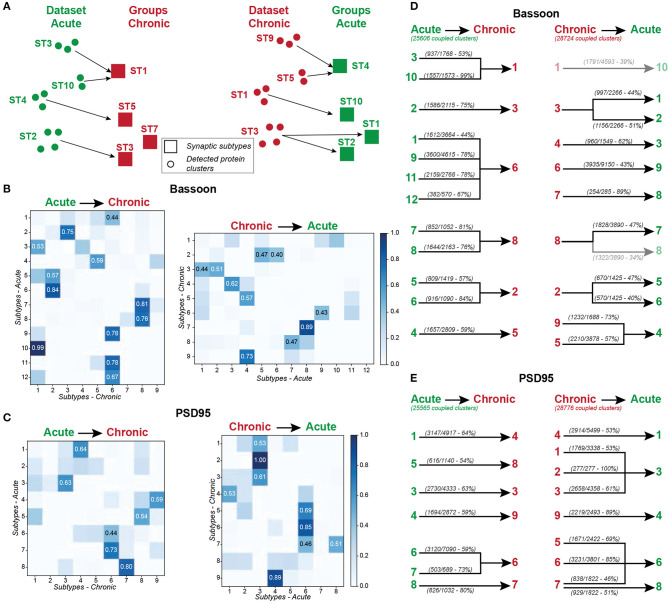
Mapping the synaptic subtypes that emerged from the acute and chronic treatments. **(A)** Schematic representation of the mapping approach. Each detected protein cluster of the acute treatment experiments (Dataset Acute, green circles) is assigned to the most similar (smallest Euclidean distance) synaptic subtype of the chronic treatment experiment (Groups Chronic, red squares) and vice versa. **(B,C)** Results of the mapping analysis, showing the percentage of clusters for Bassooon **(B)** and PSD95 **(C)** from each subtypes of one experiment (y- axis, Acute: left, Chronic: right) that are assigned to each subtype of the other experiment (x-axis, Chronic: left, Acute: right). The most prominent association (proportion above 0.4) between groups are highlighted. **(D,E)** Results of the mapping experiments for Bassoon **(D)** and PSD95 **(E)** showing all projections for which more than 40% of the protein clusters belonging to one synaptic subtype of the acute (left) or chronic (right) treatment experiment where mapped into a specific subtype of the other experiment (Chronic: left, Acute: right). The majority of the synaptic subtypes of one experiment exhibit one major connection (more than 40% of the protein clusters are associated) to one group of the other experiment.

**Figure 9:**
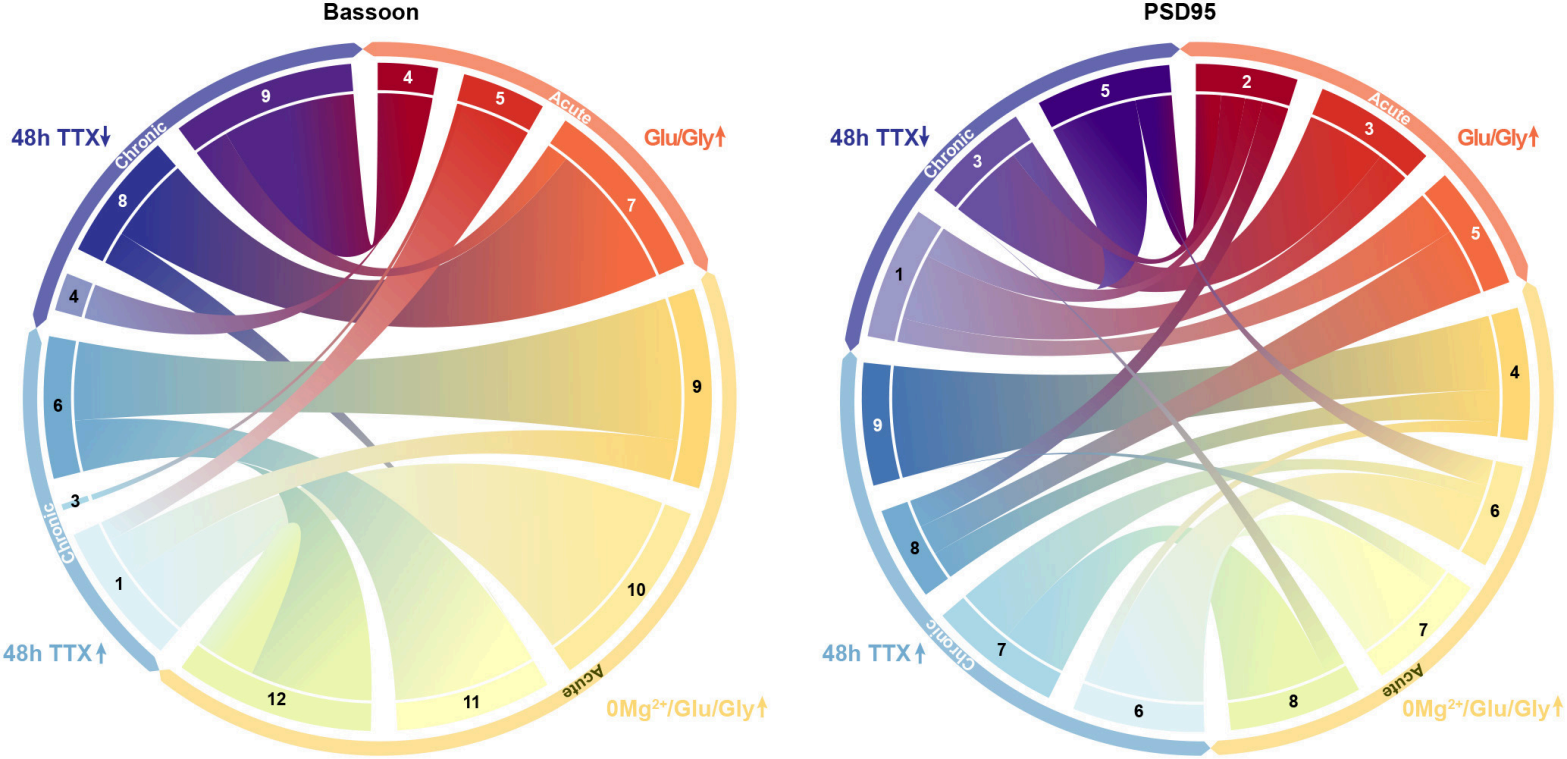
Association between synaptic subtypes depending on their prevalence in acute and chronic treatments. Synaptic subtypes that are increasing following acute treatment of 0Mg^2+^/Gly/Bic (yellow) are strongly associated with the ones increasing following a 48 h chronic TTX treatment (blue). Synaptic subtypes increasing following an acute treatment of Glu/Gly (red/orange) are mostly linked with subtypes decreasing following a 48 h chronic TTX treatment (violet) for both Bassoon (left) and PSD95 (right). The magnitude of the links between each synaptic subtype was determined from the results of the mapping experiments shown in Figures 8B,C.

In the face of this diversity, we asked whether certain synaptic subtypes emerged across the acute and chronic treatments (Supplementary Figure 28). We projected all instances (detected protein clusters) of one dataset (e.g. acute treatment) into the groups (synaptic subtypes) determined from the other dataset (e.g. chronic treatment) and vice versa (Figure 8A). We next computed the proportion of the protein clusters of one dataset that were associated to each synaptic subtype of the other dataset (Figures 8B,C). This approach shows strong correspondence between some groups, while others are unique to an experimental paradigm (Figures 8D,E). For example, for 7 of the 12 groups identified in the acute treatment experiment, more than 75% of their detected Bassoon clusters can be associated with a single group in the chronic treatment experiment (Figure 8B, ST_*acute*_2, 7-11). A predominant subtype emerges in the chronic inhibition experiment (ST_*chronic*_6, 32% of all coupled Bassoon clusters), which can be represented by 4 subgroups in the acute stimulation experiment (ST_*acute*_1, 9, 11, 12) (Figures 8B,D). In addition to the observed similarity in morphological and coupling properties between ST_*chronic*_6 and ST_*acute*_1, 9, 11, 12, we note that all these subgroups are favored by the treatments that typically induce synaptic potentiation (cLTP stimulation and chronic TTX application) (Figures 5, 7, 9, favored by 0Mg^2+^/Gly/Bic : yellow, favored by 48 h TTX : blue). A similar association between both experiments is observed for strongly coupled PSD95 clusters for which ST_*acute*_4, 6, 7, 8 & ST_*chronic*_6, 7, 9 are highly correlated (Supplementary Figure 28) and are favored by cLTP or chronic TTX stimuli (Figures 5, 7, 9, favored by 0Mg^2+^/Gly/BIC : yellow, favored by 48 h TTX : blue). Similarly, a correlation, although less strong, is observed between the subtypes promoted by the LTD paradigm and those decreasing following chronic TTX treatment (Figure 9, favored by Glu/Gly : red/orange, decreased by 48 h TTX : violet).

Additionally, the unsupervised machine learning approach identified subtypes that are specific to one stimulation paradigm, generally encompassing a small proportion of the overall detected clusters, and that are not mapped between experiments. This is the case for the Bassoon ST_*acute*_12 and PSD95 ST_*acute*_7 which describe small, eccentric, and strongly coupled protein clusters and are solely found following a cLTP stimulus (Figures 5E,F, 8B,C). These subtypes would likely have been overlooked by conventional rule-based analysis, as they describe only a small proportion of the data of a single stimulus paradigm.

Thus, the unsupervised grouping approach used to describe synaptic remodeling shows strong correlation between synaptic subtypes of the acute and homeostatic plasticity paradigms. It highlights strong similarities in the effect of cLTP and 48 h TTX treatment on synaptic organization and architecture.

## 4. Discussion

We implemented a high-throughput analysis framework based on statistical object distance analysis, pySODA, to investigate the diversity of synaptic remodeling at the population level and discriminate distinct characteristics of synaptic protein clusters. We chose pySODA to detect pairs of synaptic protein clusters, as this approach was shown to be less dependent on labeling density, optical resolution, and signal intensity. Importantly, it provides an unbiased selection of clusters, ensuring the analysis of nearly all detectable synapses. Our study examined the activity-dependent remodeling of the nanoscale architecture of the active zone and the postsynaptic density by characterizing the interactions between pre- (RIM 1/2, Bassoon) and postsynaptic (PSD95, Homer1c) protein pairs in cultured neuronal circuits. We combined pySODA with unsupervised machine learning approaches to enrich the characterization of activity-dependent changes in synaptic protein organization based on the coupling and morphological properties of PSD95 and Bassoon synaptic clusters. This high-throughput analysis of STED images allowed us to examine the synaptic re-organization at the population level (between 12,500 and 25,000 protein clusters depending on the condition) in response to treatments inducing acute or homeostatic plasticity.

We first addressed whether a chemical LTP-inducing stimulus (0Mg^2+^/Gly/Bic) affects the organization of these scaffold elements within their synaptic compartment (pre- or postsynaptic). We show that Bassoon and RIM1/2 are strongly coupled and that neither their coupling probability nor their mean coupling distance are affected by the LTP-inducing stimulus. Conceivably, the presynaptic pair may still undergo activity-dependent re-organization at a scale that the resolution of this approach cannot detect. For example, Glebov et al. (2017) implemented a FRET-based measurement to show unclustering of Bassoon upon chronic TTX treatment, an effect that was not detectable with STORM.

In contrast, we show that the same stimulus reduces the coupling probability and increases the mean coupling distance between Homer1c and PSD95. These results indicate that the extent of remodeling between the postsynaptic pair is more pronounced as compared to that of the presynaptic pair. While the relationship between Bassoon and RIM1/2 during synaptic plasticity has not been examined previously, it has been shown that activity inducing stimuli can induce a rapid declustering of Homer1c (Okabe et al., 2001; Kuriu et al., 2006) as well as PSD95 (Steiner et al., 2008; Fukata et al., 2013) at the postsynaptic compartment. Our data on the decreasing coupling properties of PSD95 - Homer1c during synaptic stimulation are thus consistent with these studies.

When looking at the relationship between pre- and post-synaptic partners, we found that the cLTP stimulus has opposite effects on the spatial organization of Homer1c and PSD95 to Bassoon. Indeed the coupling probability of Homer1c - Bassoon decreases (and coupling distance increases), whereas it increases for Bassoon - PSD95 (and coupling distance decreases). A pool of PSD95 was reported to leave the synapse upon synaptic stimulation (Steiner et al., 2008; Doré et al., 2014), while we observed an increase in the coupling probability between PSD95 and Bassoon. We can speculate that a loosely coupled pool of PSD95 leaves the postsynaptic area upon stimulation, and that the remaining pool exhibits an increased degree of coupling. The further activity-dependent reduction in coupling probability of Homer1c to both PSD95 and Bassoon suggests that Homer1c has a weaker association with pre- and postsynaptic scaffolds upon LTP-inducing stimulation. The activity-dependent redistribution of Homer1c has not been clearly established (Okabe et al., 2001; Tao-Cheng et al., 2014; Lagache et al., 2018). It is interesting to note that Homer1c was shown to associate with Shank and GTPase dynamin-3 to form a complex linking the PSD with the clathrin endocytotic zone, which is necessary for endocytosis of AMPA receptors, a process occurring mainly on the periphery of the PSD (Lu et al., 2007). Hence, the activity-dependent uncoupling of Homer1c from the postsynaptic area may regulate AMPA receptors endocytosis supporting synaptic plasticity (Petrini et al., 2009).

Tang et al. (2016) nicely showed, using super-resolution microscopy, that LTP- and LTD-inducing stimuli produce opposite effects on the alignment of transsynaptic nanocolumns in dissociated hippocampal circuits. Using pySODA, we measured an opposite effect of these stimuli on the coupling probability of PSD95 and Bassoon at the population level. The cLTP condition exhibited a larger proportion of highly coupled pairs, as compared to controls, while in the LTD condition, weakly coupled pairs were more prevalent. Thus, these observed changes in the coupling probability could serve as a readout of synaptic re-organization at the population level representing early phases of LTP and LTD. Chronic inhibition of neuronal activity, known to induce synaptic upscaling (Turrigiano et al., 1998), also resulted in increased coupling probability between PSD95 and Bassoon. On the other hand, the TTX treatment did not significantly change the mean coupling distance, contrasting with the effect of the LTP stimulus, which may reflect a different mechanism of potentiation.

Our results are consistent with previous reports of an increase in PSD95 area associated with acute or homeostatic synaptic plasticity (MacGillavry et al., 2013; Tang et al., 2016). They additionally demonstrate an increase in the organization of pre- and postsynaptic scaffolds as reported by recent studies (Tang et al., 2016; Hruska et al., 2018; Crosby et al., 2019). Furthermore, we observed that highly coupled Bassoon and PSD95 clusters exhibit a wide range of sizes and eccentricities, which may reveal different strategies employed by synapses to express plasticity, that include the modulation of PSD shape (Stewart et al., 2005), increase in spine size (Matsuzaki et al., 2004), *de novo* synapse formation (Kwon and Sabatini, 2011), nanometric re-organization of transsynaptic nanocolumns (Tang et al., 2016) or spine organelle content (Borczyk et al., 2019).

Using the dimensionality reduction technique UMAP combined with hierarchical grouping, we identified a broad range of synaptic subtypes based on their morphological and coupling characteristics. The plasticity-inducing treatments changed the proportions of these subtypes in various ways, yet, remarkably, common subtypes emerged for conditions causing synaptic potentiation as well as for depression. Thus, the two independent methods of analysis presented here, with no prior knowledge of the conditions or pre-determined criteria of synaptic features, converge on common features of synaptic properties encoding synaptic potentiation or depression.

We suggest that the expanded palette of synaptic features revealed by our unbiased approach, focusing on large numbers of synapses, provides a basis to further explore the widely diverse molecular mechanisms of synaptic plasticity. In our study, we exploited dissociated cultured hippocampal neurons, a preparation frequently used to highlight molecular traits of LTP, LTD, or synaptic scaling (Tang et al., 2016; Glebov et al., 2017). This approach does not, however, provide the physiological information obtained from recordings of paired neurons in brain slice. Combining both types of information will be important and will require the ability to monitor a large number of live synapses with combined readouts of synaptic proteins and functional activity. Future studies may profit from our analysis framework to investigate the diversity of the synaptic protein organization across various brain regions (Broadhead et al., 2016), during aging (VanGuilder et al., 2011), or even across species (Ryan and Grant, 2009) to learn more on the diversity of synaptic protein organization supporting learning and memory.

## Data Availability Statement

The analysis routine generated for this study can be found online: https://github.com/FLClab/pySODA and https://github.com/FLClab/MultidimSynapticProteins. The dataset generated for this study can be obtained from the corresponding author upon reasonable request.

## Ethics Statement

The animal study was reviewed and approved by the Animal Care Committee of Université Laval.

## Author Contributions

TW and BR performed STED imaging. TW prepared samples. RB, AB, AD, and TW wrote the analysis routine. TW, AB, AD, RB, and FL-C analyzed the data. TW and FL-C planned the STED experiments. AB, TW, and FL-C designed and implemented the machine learning-based analysis. TW, AB, PDK, and FL-C wrote the manuscript. PDK and FL-C co-supervised the project.

## Conflict of Interest

The authors declare that the research was conducted in the absence of any commercial or financial relationships that could be construed as a potential conflict of interest.
