# Supplementary material for: Activity-Dependent Remodeling of Synaptic Protein Organization Revealed by High Throughput Analysis of STED Nanoscopy Images
Source: Front Neural Circuits. 2020 Oct 15;14:57. doi: 10.3389/fncir.2020.00057 (PMC7594516; doi:10.3389/fncir.2020.00057)
Supplement: Supplementary file 1 [file Data_Sheet_1.pdf]

Supplementary Material

1 SUPPLEMENTARY TABLES AND FIGURES

1.1 Figures

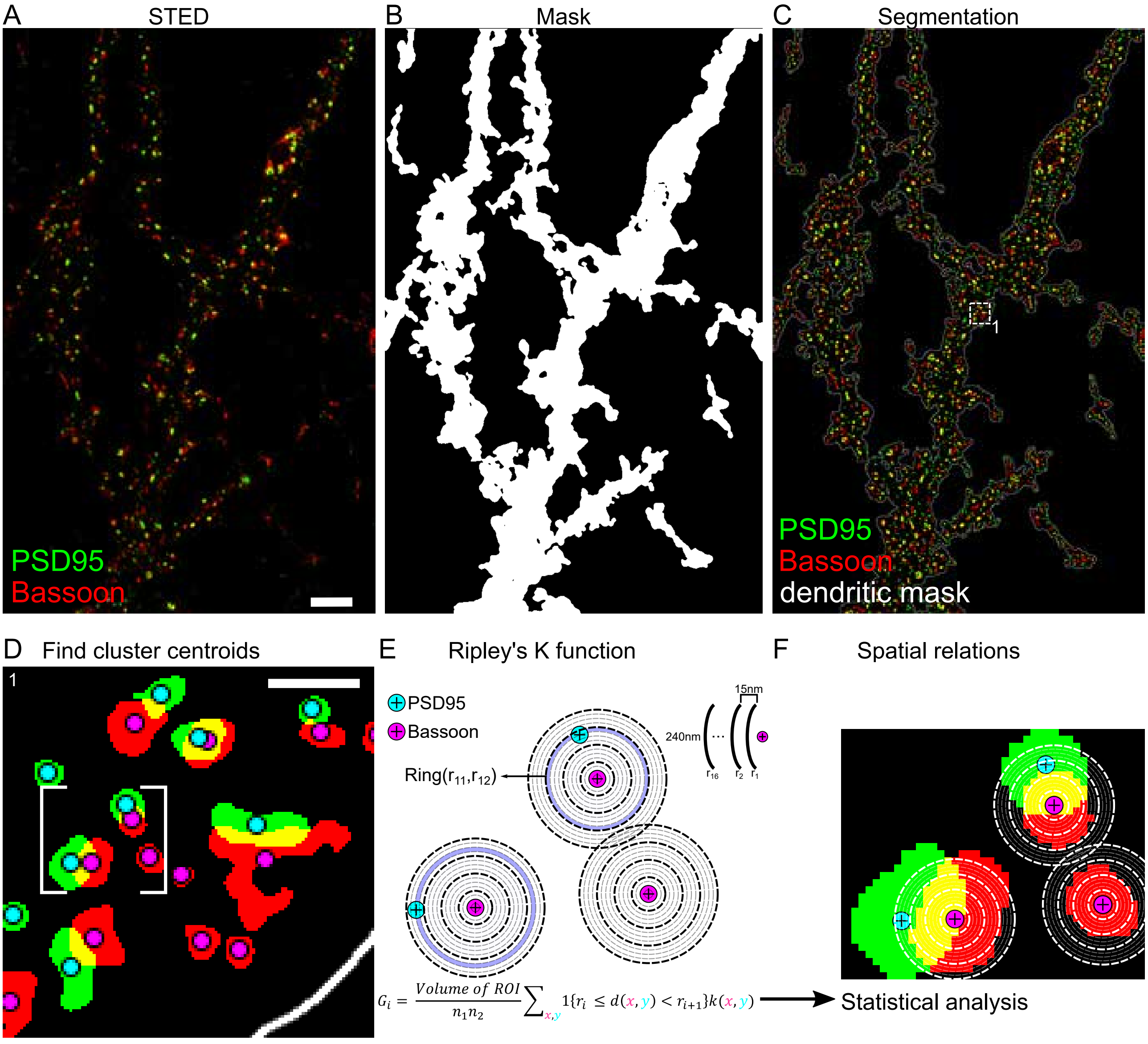

Caption is on the next page

Figure S1: pySODA workflow. A) Two-color STED image of Bassoon - STAR635P (red) and PSD95 - Alexa594 (green). B) Foreground mask generated by a gaussian kernel convolution on the sum of both STED channels, followed by an intensity threshold on the convoluted image (see Methods). C) Wavelet segmentation of Bassoon (red) and PSD95 (green) clusters within the foreground mask (white line). D) Magnification of the region identified with a white square on the segmentation map in C showing Bassoon (red) and PSD95 (green) clusters overlaid with their corresponding weighted centroids (Bassoon: magenta and PSD95: cyan). E) Weighted centroids of the synaptic clusters shown in the region identified with a white bracket in D. 16 concentric rings around the centroids and spaced by 15 nm were used to calculate the G vector of the Ripley's K function. F) Concentric rings around Bassoon clusters overlaid with the wavelet segmentation for the region identified with a bracket in D. Scale bars: A) 5 μm, D) 500 nm.

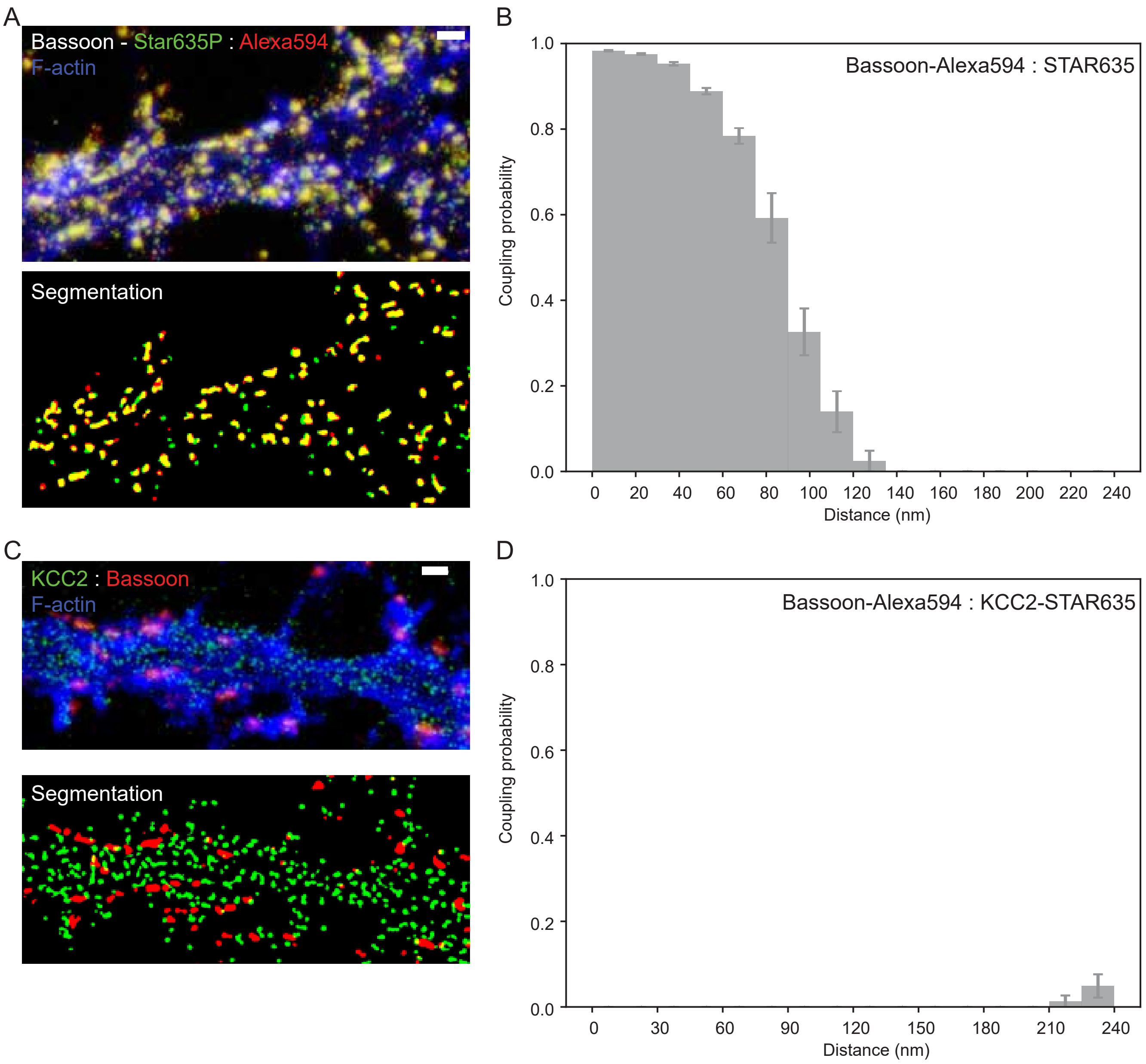

Figure S2: pySODA analysis of strongly colocalized and randomly distributed protein clusters. A) *Top*: 3-Channel STED and confocal image of Bassoon (STED) labeled concurrently with two secondary antibodies tagged with different fluorophores (Alexa594 (red) and STAR635P (green)) and F-actin (Confocal, Phalloidin - Alexa488 (blue)). *Bottom*: Wavelet segmentation of Bassoon - Alexa594 (red) and Bassoon-STAR635P (green) clusters. The overlap between segmented clusters is shown in yellow. B) Histogram of the coupling probability at a given distance for Bassoon - Alexa594/STAR635P (n = 17). Each 15 nm bin represents the average coupling probability calculated from the individual images with standard error. C) *Top*: 3-Channel STED and confocal image of KCC2 - STAR635P (STED, green), Bassoon-Alexa594 (STED, red) and F-actin - Phalloidin - Alexa 488 (confocal, blue). *Bottom*: Wavelet segmentation of Bassoon - Alexa594 (red) and KCC2 - STAR635P (green) clusters. D) Histogram of the coupling probability at a given distance for Bassoon - KCC2 (n = 17). Each 15 nm bin represents the average coupling probability calculated from the individual images with standard error. A, C) To better distinguish low intensity clusters combined with the F-actin signal, a log intensity scale was used for the raw images. B, D) Number of images of independent neurons (n). Scale bars 500 nm.

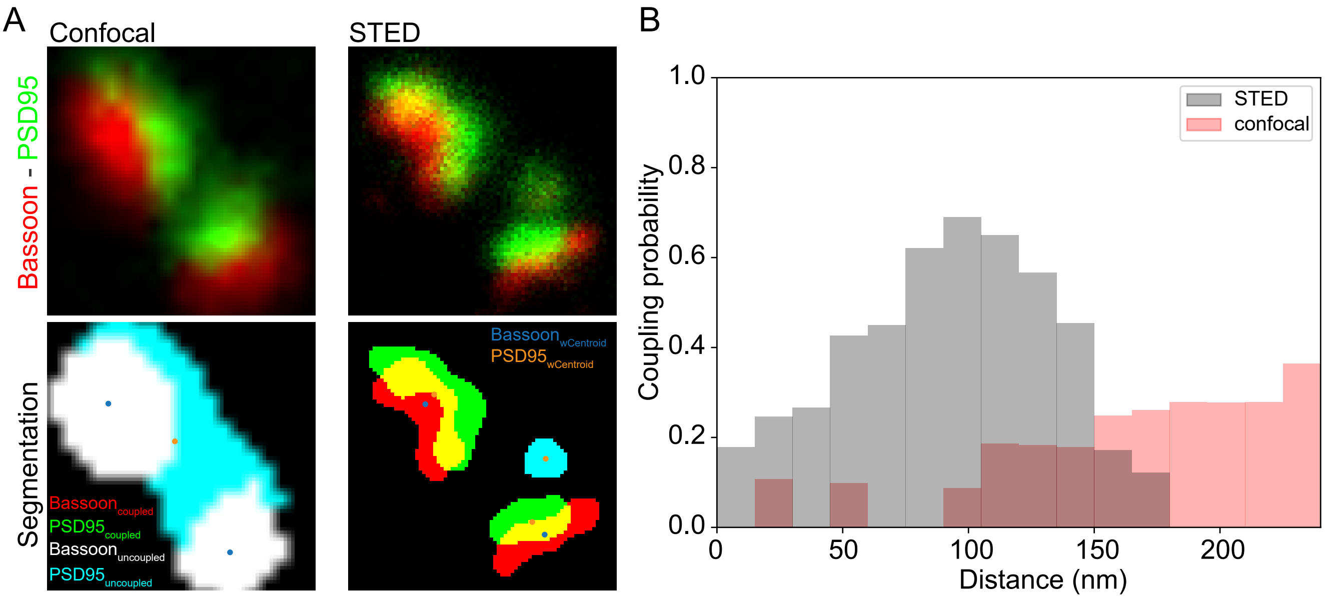

Figure S3: pySODA analysis on confocal or STED images of PSD95 and Bassoon. A) Example confocal and STED images *(top)* of the protein Bassoon labeled with STAR 635P (red) and PSD95 tagged with Alexa 594 (green). The wavelet segmentation followed by SODA *(below)* shows that both the position of the weighted centroids and the coupling between the protein clusters are strongly affected by the resolution of the microscope. B) Coupling probability - Distance histogram reveals that confocal resolution is insufficient to calculate the association of PSD95 and Bassoon clusters. n = 8 neurons.

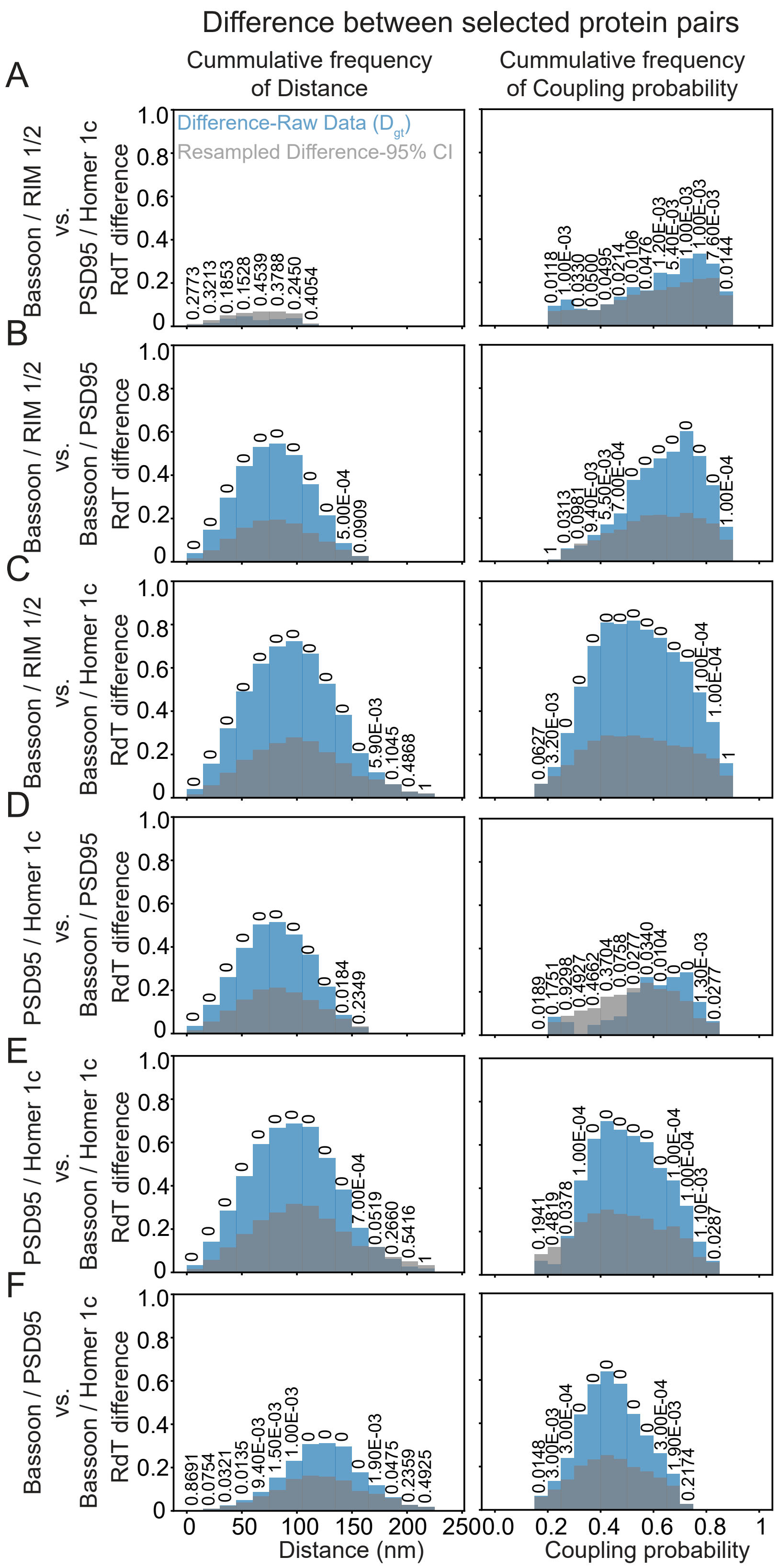

Caption is on the next page

Figure S4: Statistical analysis using a randomization test for the comparison between the cumulative frequencies of the coupling distance *(left)* and the coupling probability *(right)* for the synaptic scaffold proteins pairs shown in Figure 2. For all panels, the absolute difference between the mean values of the raw data (D_{gt}, see Methods) is depicted in blue. The 95% confidence interval (CI) of the randomization test difference (D_{rand}, see Methods) is obtained from 10 000 repetitions and shown in grey. We verify the null hypothesis that two conditions belong to the same distribution when the resampled D_{rand} is greater than D_{gt} considering a 95% CI. The comparison is made for A) Bassoon - RIM1/2 versus PSD95 - Homer1c, B) Bassoon - RIM1/2 versus Bassoon - PSD95, C) Bassoon - RIM1/2 versus Bassoon - Homer1c, D) PSD95 - Homer1c versus Bassoon - PSD95, E) PSD95 - Homer1c versus Bassoon - Homer1c, and F) Bassoon - PSD95 versus Bassoon - Homer1c.

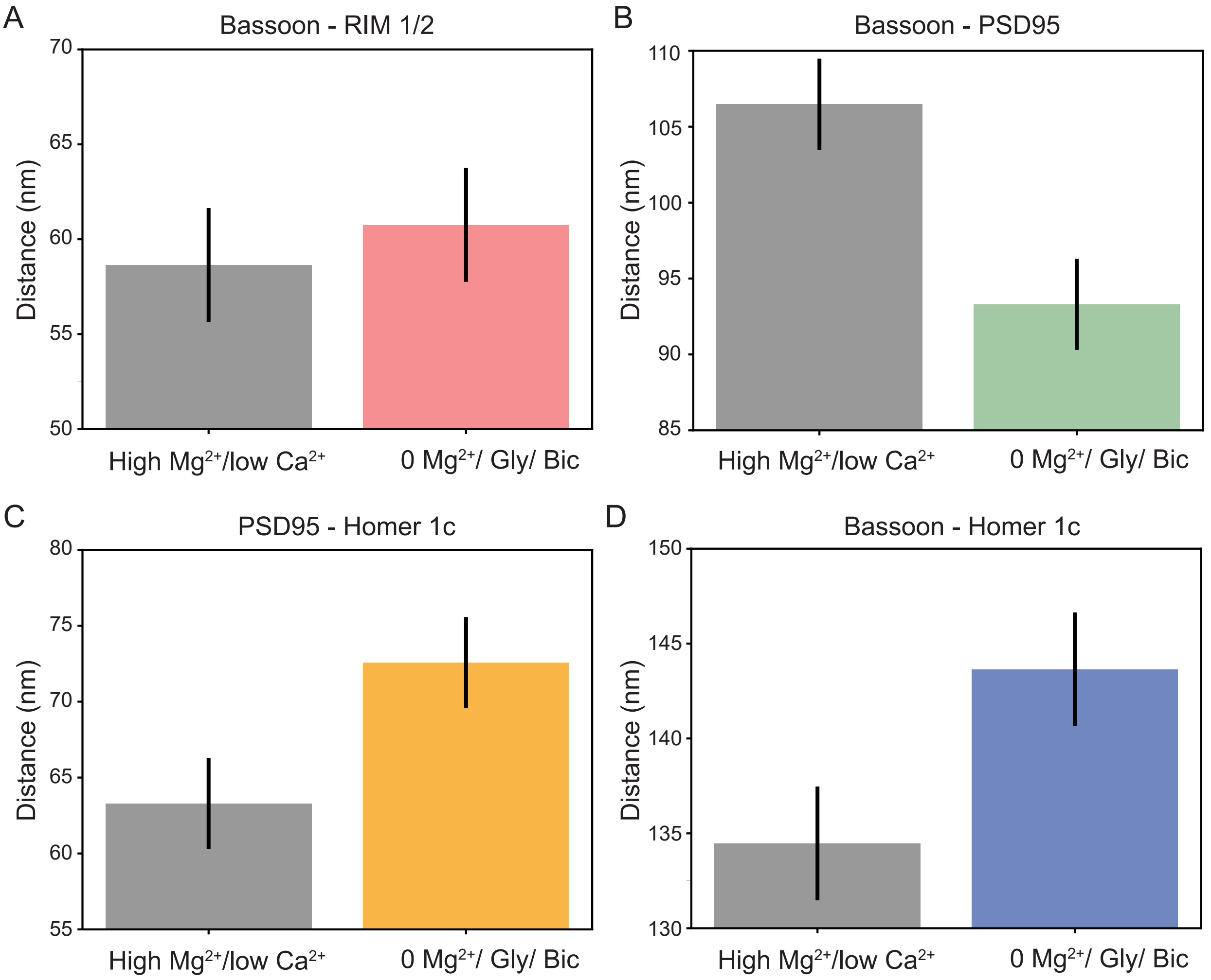

Figure S5: Mean coupling distances between synaptic protein pairs for different levels of synaptic activity. Bar graphs show the mean coupling distance measured for all coupled clusters for the low-activity high Mg^{2+}/low Ca^{2+} condition (grey) and following the LTP-inducing stimulus $0Mg^{2+}$/Gly/Bic (the color code corresponds to the one used in Figure 2). A) Bassoon - RIM1/2, B) Bassoon - PSD95, C) PSD95 - Homer1c and D) Bassoon - Homer1c. The error bars correspond to the calculated precision error of 3 nm for the localization of the weighted centroids from the raw STED images (see Methods).

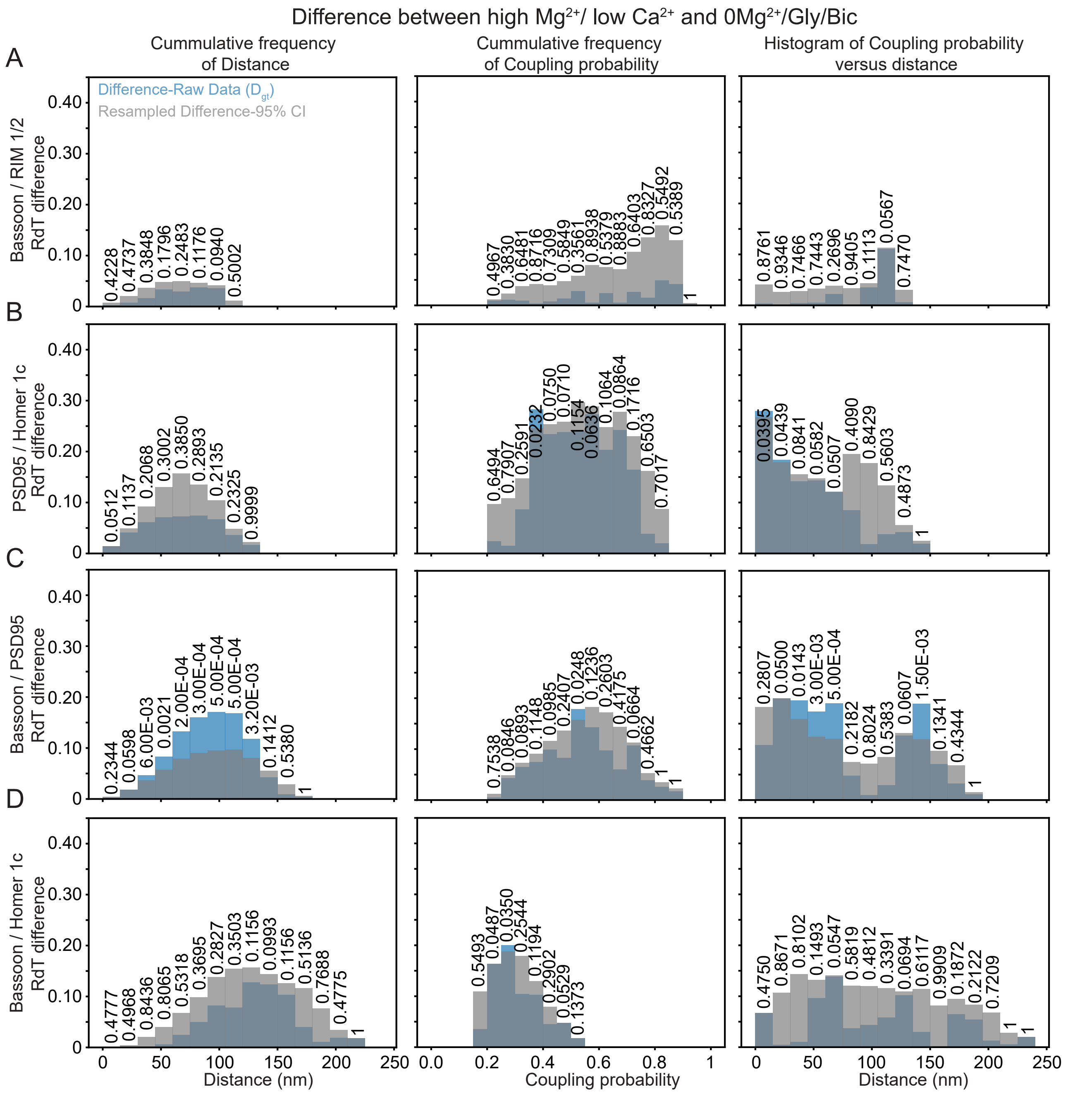

Figure S6: Statistical analysis using a randomization test for the comparison between low and high neuronal activity for the cumulative frequencies of the coupling distance *(left)*, the coupling probability *(middle)*, and the histogram of mean coupling probability at a given distance *(right)* that are shown in Figure 3. For all panels, the absolute difference between the mean values of raw data (D_{gt}, see Methods) is depicted in blue. The 95% confidence interval of the randomization test difference (D_{rand}, see Methods) is obtained from 10 000 repetitions and shown in grey. We verify the null hypothesis that two conditions belong to the same distribution when the resampled D_{rand} is greater than D_{gt} considering a 95% CI. The comparison is made between high Mg^{2+}/low Ca^{2+} and 0Mg^{2+}/Gly/Bic for the synaptic protein pairs A) Bassoon - RIM1/2, B) PSD95 - Homer1c, C) Bassoon - PSD95 and D) Bassoon - Homer1c.

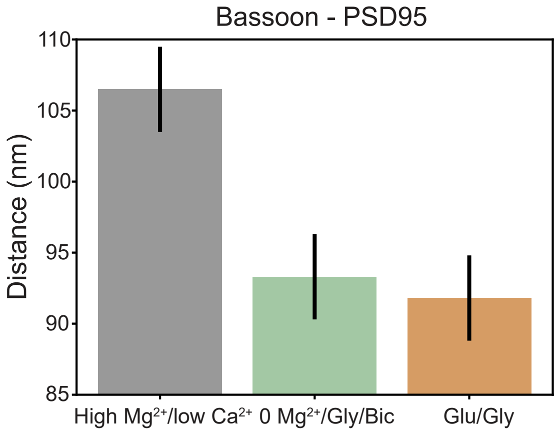

Figure S7: Mean distance measured between coupled Bassoon and PSD95 cluster pairs (high Mg^{2+}/low Ca^{2+} (grey), $0Mg^{2+}$/Gly/Bic (green), and Glu/Gly (orange)). The error bars correspond to the calculated precision error of 3 nm for the localization of the weighted centroids from the raw STED images (see Methods).

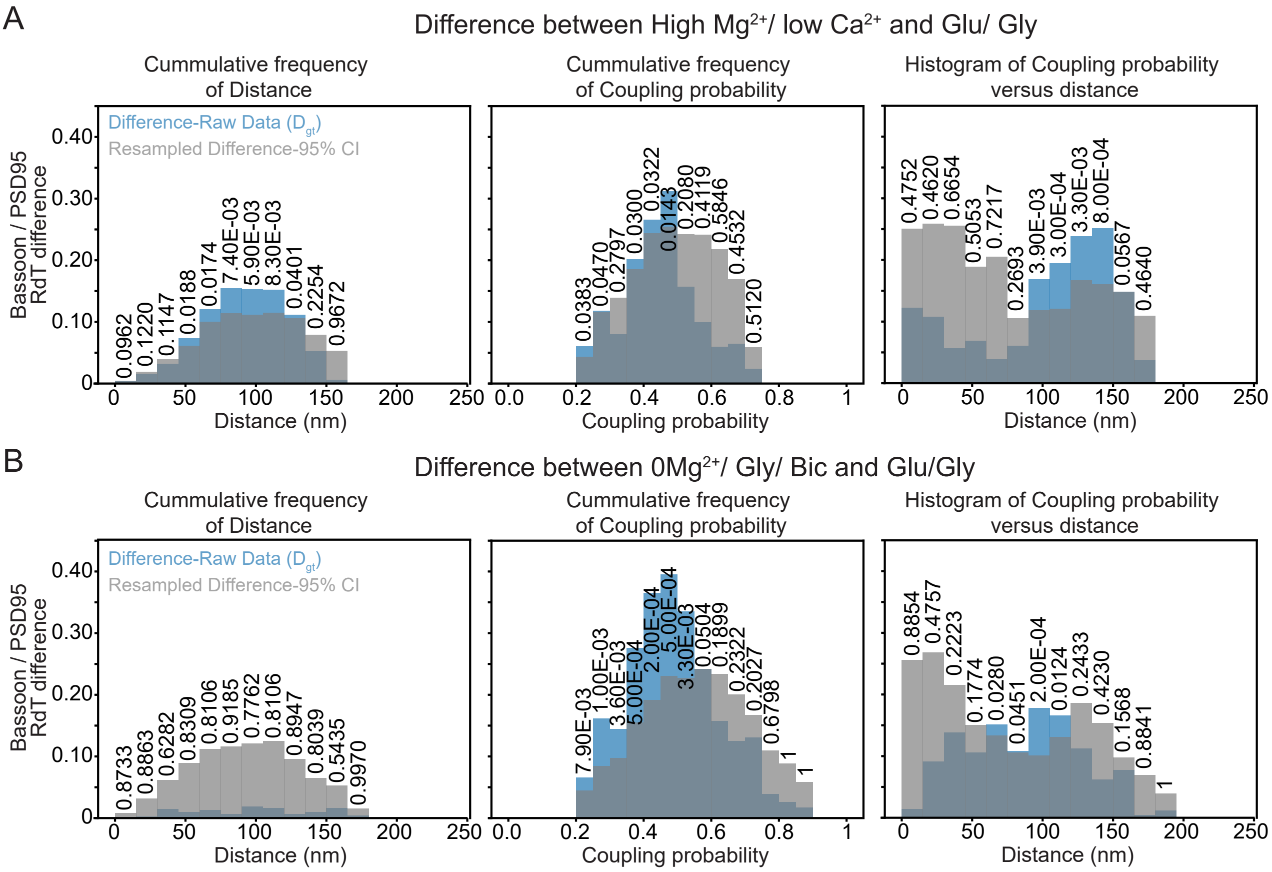

Figure S8: Statistical analysis using a randomization test for the comparison between different stimulation paradigms of the scaffold protein pair Bassoon and PSD95. The randomization test is performed for the cumulative frequencies of the distance *(left)*, the coupling probability *(middle)*, and the histogram of mean coupling probability at a given distance *(right)*. For all panels, the absolute difference between the mean values of raw data (D_{gt}, see Methods) is depicted in blue. The 95% confidence interval of the randomization test difference (D_{rand}, see Methods) is obtained from 10 000 repetitions and shown in grey. We verify the null hypothesis that two conditions belong to the same distribution when the resampled D_{rand} is greater than D_{gt} considering a 95% CI. The comparison is made for A) high Mg^{2+}/low Ca^{2+} versus Glu/Gly, and B) $0Mg^{2+}$/Gly/Bic versus Glu/Gly.

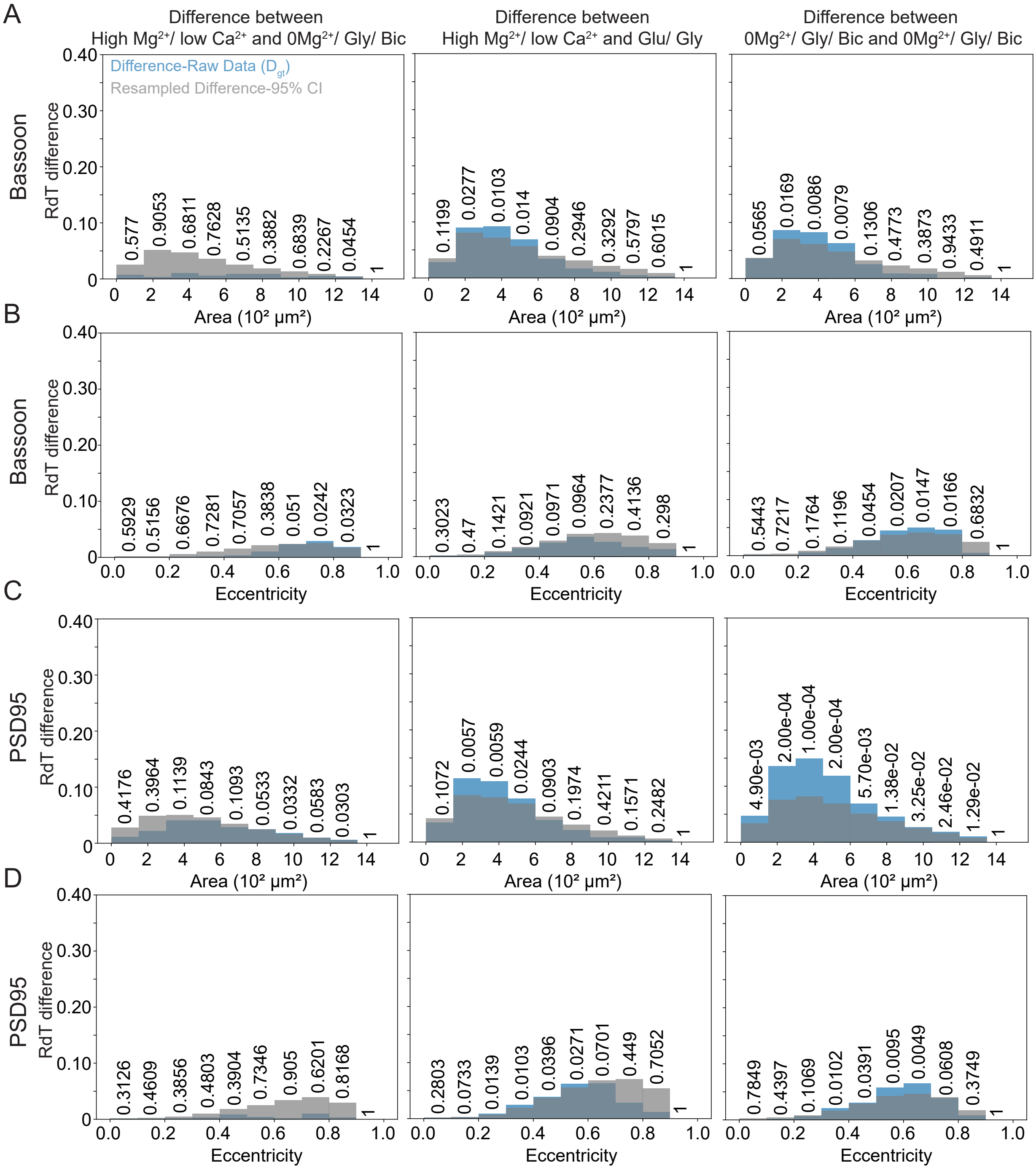

Figure S9: Statistical analysis using a randomization test for the comparison between the different acute synaptic activity paradigms for the cumulative frequencies of the area *(left)*, the eccentricity *(middle)* are shown in Figure 4. For all panels, the absolute difference between the mean values of raw data (D_{gt}, see Methods) is depicted in blue. The 95% confidence interval of the randomization test difference (D_{rand}, see Methods) is obtained from 10 000 repetitions and shown in grey. We verify the null hypothesis that two conditions belong to the same distribution when the resampled D_{rand} is greater than D_{gt} considering a 95% CI. The comparison is made between high Mg^{2+}/low Ca^{2+}, 0Mg^{2+}/Gly/Bic and Glu/Gly for the coupled protein clusters of A) and B) Bassoon and C) and D) PSD95.

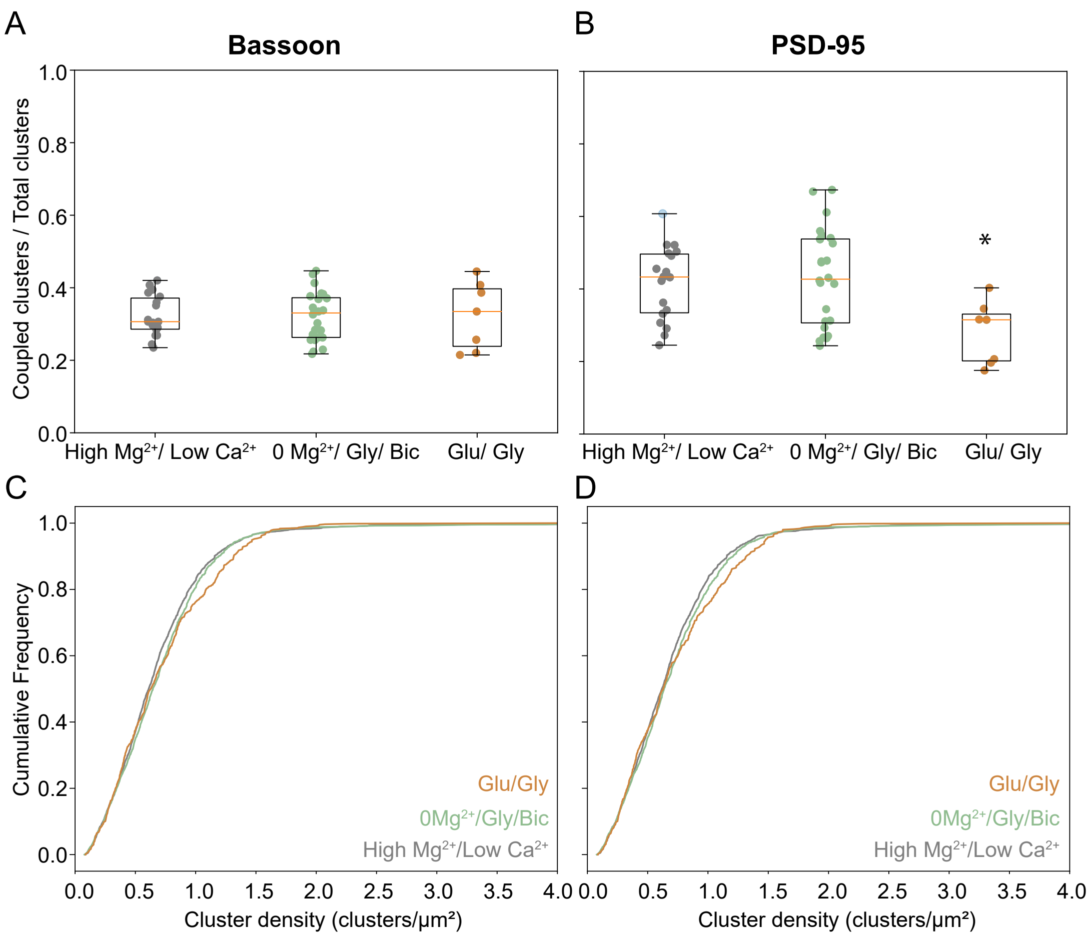

Figure S10: The proportion of coupled PSD95 clusters affected by an LTD-inducing stimulus. Ratios of coupled/total clusters for the acute plasticity experiment showing that the proportion of coupled clusters is not affected by the stimulation for A) Bassoon, while it is significantly reduced by a Glu/Gly stimulation for B) PSD95 (ANOVA: p = 0.0150, t-test: p = 0.0047). Cumulative frequency curves showing that the density of coupled cluster is not affected by the short (2 min Glu/Gly or 10 min 0Mg^{2+}/Gly/Bic) acute stimulations for both proteins. Measured for coupled C) Bassoon and D) PSD95 clusters in high Mg^{2+}/low Ca^{2+}(black, n = 18), 0Mg^{2+}/Gly/Bic (green, n = 24), and Glu/Gly (orange, n = 7).

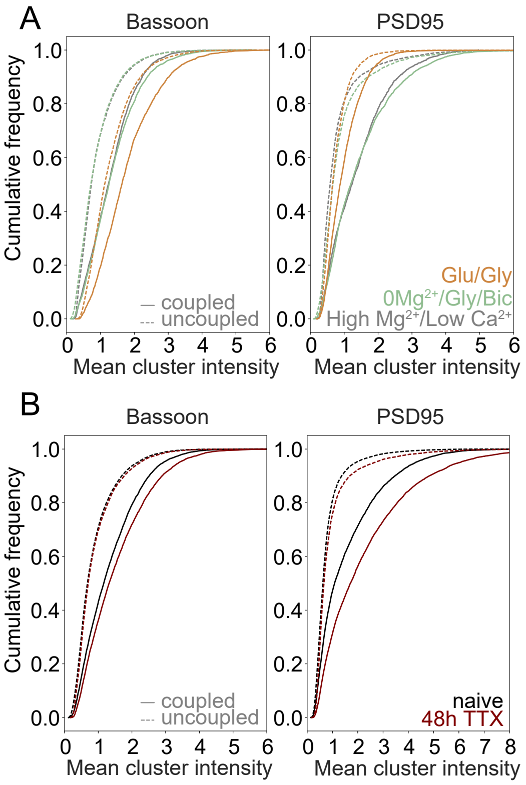

Figure S11: Cumulative frequency curves of the mean cluster intensity for coupled and uncoupled Bassoon and PSD95 clusters. A) For the acute stimulation paradigm, the mean intensity of coupled clusters (solid line) is higher compared to uncoupled clusters (dashed line) for both Bassoon and PSD95 regardless of the condition for both proteins. The mean cluster intensity of coupled and uncoupled Bassoon clusters is increased by the Glu/Gly (orange) stimulation compared to the low activity high Mg^{2+}/low Ca^{2+} condition (dark grey). For coupled PSD95 clusters, the mean cluster intensity is decreased by the Glu/Gly stimulation, while there seems to be no effect on uncoupled clusters. B) For the homeostatic plasticity paradigm, the mean intensity of coupled clusters (solid line) is higher compared to uncoupled clusters (dashed line) for both Bassoon and PSD95 regardless of the condition. For both proteins, 48h chronic TTX treatment results in an increased mean cluster intensity. Since quantitative fluorescence intensity measurements, especially in the context of super-resolution microscopy (Ta et al., 2015), are known to be strongly affected by variation of molecular brightness and are compromised by environmental factors (e.g. heterogeneity of the molecular environment, sample preparation and molecular interactions), we only performed the mean cluster intensity analysis on neurons from the same preparation. (high Mg^{2+}/low Ca^{2+} - n =4, grey; $0Mg^{2+}$/Gly/Bic - n = 8, green; Glu/Gly - n = 4, orange; naive - n = 20, black; 48h TTX - n= 14, dark red)

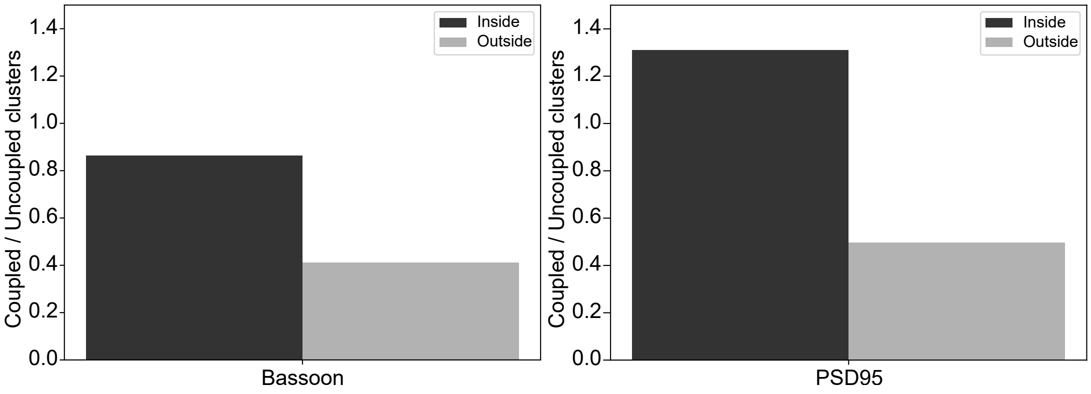

Figure S12: Proportion of coupled clusters within spines (dark grey) or on the dendritic shaft (light grey) for Bassoon (*left*) and PSD95 (*right*). Spines (1143 spines) were identified using the confocal F-actin signal (Phalloidin - Oregon Green 488) on 4 neurons of the high Mg^{2+}/low Ca^{2+} condition.

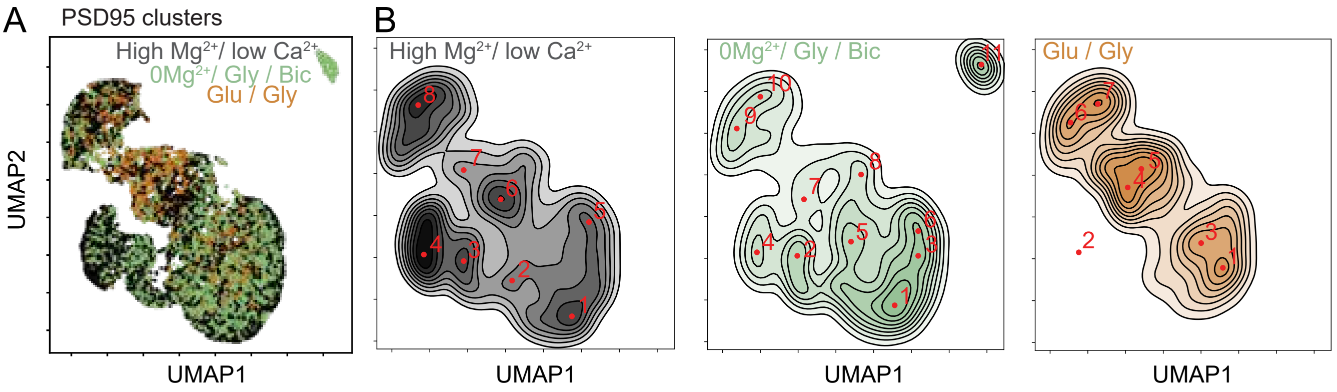

Figure S13: UMAP embedding of the coupling and morphology features for coupled PSD95 clusters. A) Scatterplot and B) corresponding kernel density estimation (KDE) maps of the UMAP embedding for high Mg^{2+}/low Ca^{2+} (black), $0Mg^{2+}$/Gly/Bic (green) and Glu/Gly (orange). Local maxima (numbers in red) identifying synaptic cluster subtypes are indicated on the KDE plots (see Methods).

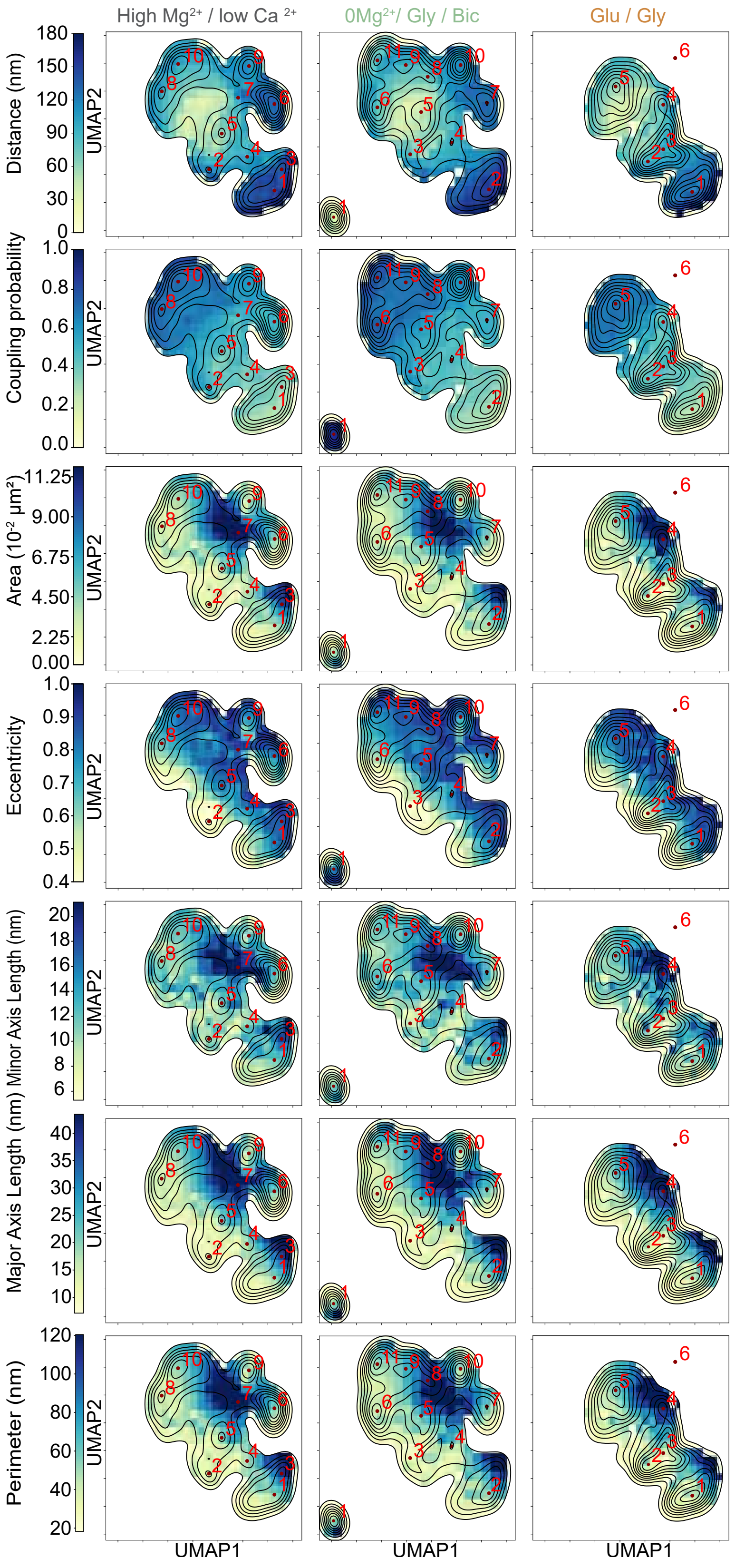

Caption is on the next page

Figure S14: KDE maps of the UMAP embedding overlaid with the color-coded distribution of coupling and morphological Bassoon cluster features. *(From top to bottom)*: distance, coupling probability, area, eccentricity, minor axis length, major axis length and perimeter. The distributions are compared for the high Mg^{2+}/low Ca^{2+} *(left)*, $0Mg^{2+}$/Gly/Bic *(middle)*, Glu/Gly *(right)* conditions. Minimum (yellow) and maximum (dark blue) values of the color-code are set for each feature: Coupling probability (min: 0, max: 1), Distance (min: 0 nm, max: 180 nm), Area (min: $0.7 \cdot 10^{-2}$ μm^2, max: $11.8 \cdot 10^{-2}$ μm^2), Eccentricity (min: 0.4, max: 1), Minor axis length (min: 5.3 nm, max: 21.1 nm), Major axis length (min: 7.1 nm, max: 43.5 nm) and Perimeter (min: 18.2 nm, max: 120.5 nm).

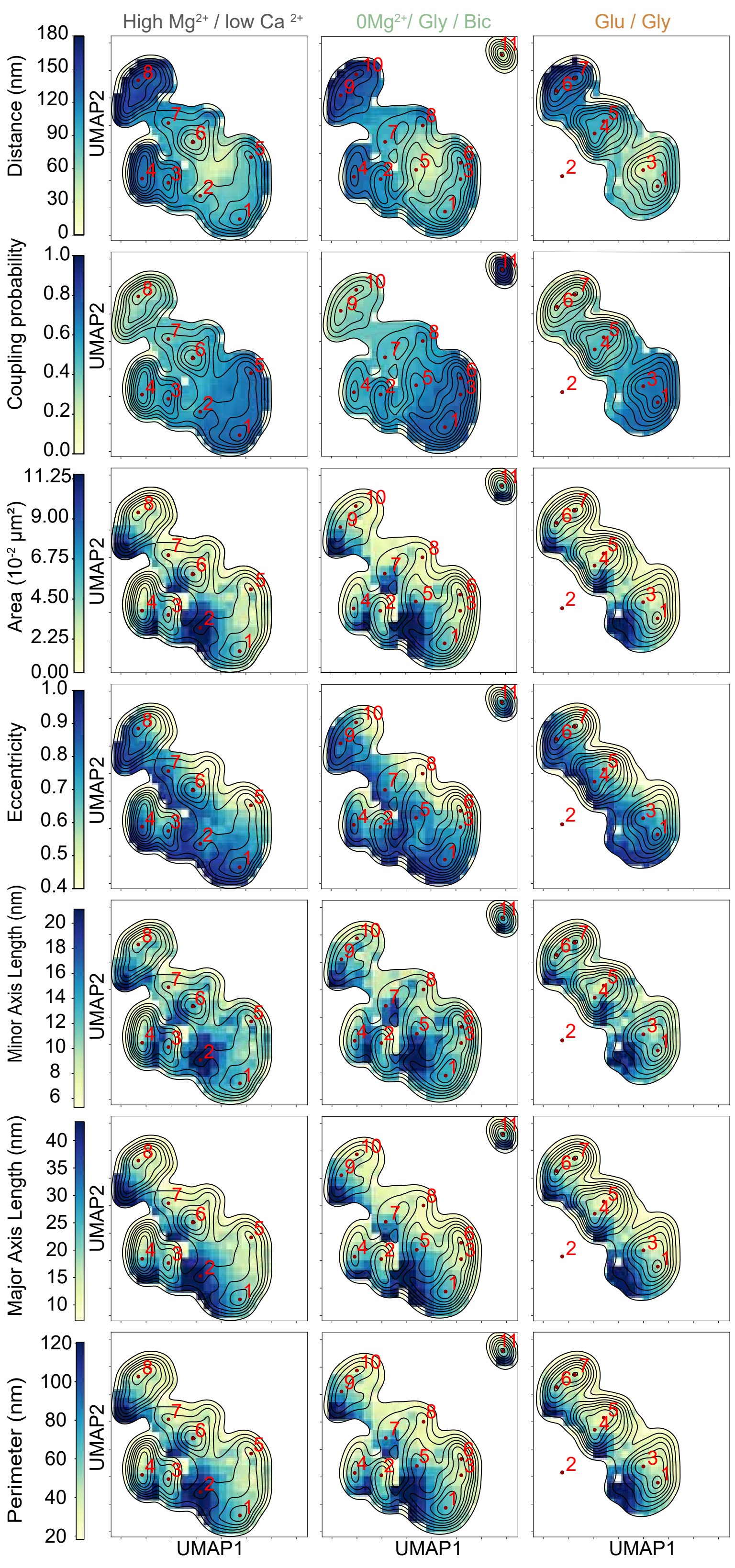

Caption is on the next page

Figure S15: KDE maps of the UMAP embedding overlaid with the color-coded distribution of coupling and morphological PSD95 cluster features. *(From top to bottom)*: distance, coupling probability, area, eccentricity, minor axis length, major axis length and perimeter. The distributions are compared for the high Mg^{2+}/low Ca^{2+} *(left)*, $0Mg^{2+}$/Gly/Bic *(middle)*, Glu/Gly *(right)* conditions. Minimum (yellow) and maximum (dark blue) values of the color-code are set for each feature: coupling probability (min: 0, max: 1), distance (min: 0 nm, max: 180 nm), area (min: $0.7 \cdot 10^{-2}$ μm^2, max: $11.8 \cdot 10^{-2}$ μm^2), eccentricity (min: 0.4, max: 1), minor axis length (min: 5.3 nm, max: 21.1 nm), major axis length (min: 7.1 nm, max: 43.5 nm) and perimeter (min: 18.2 nm, max: 120.5 nm).

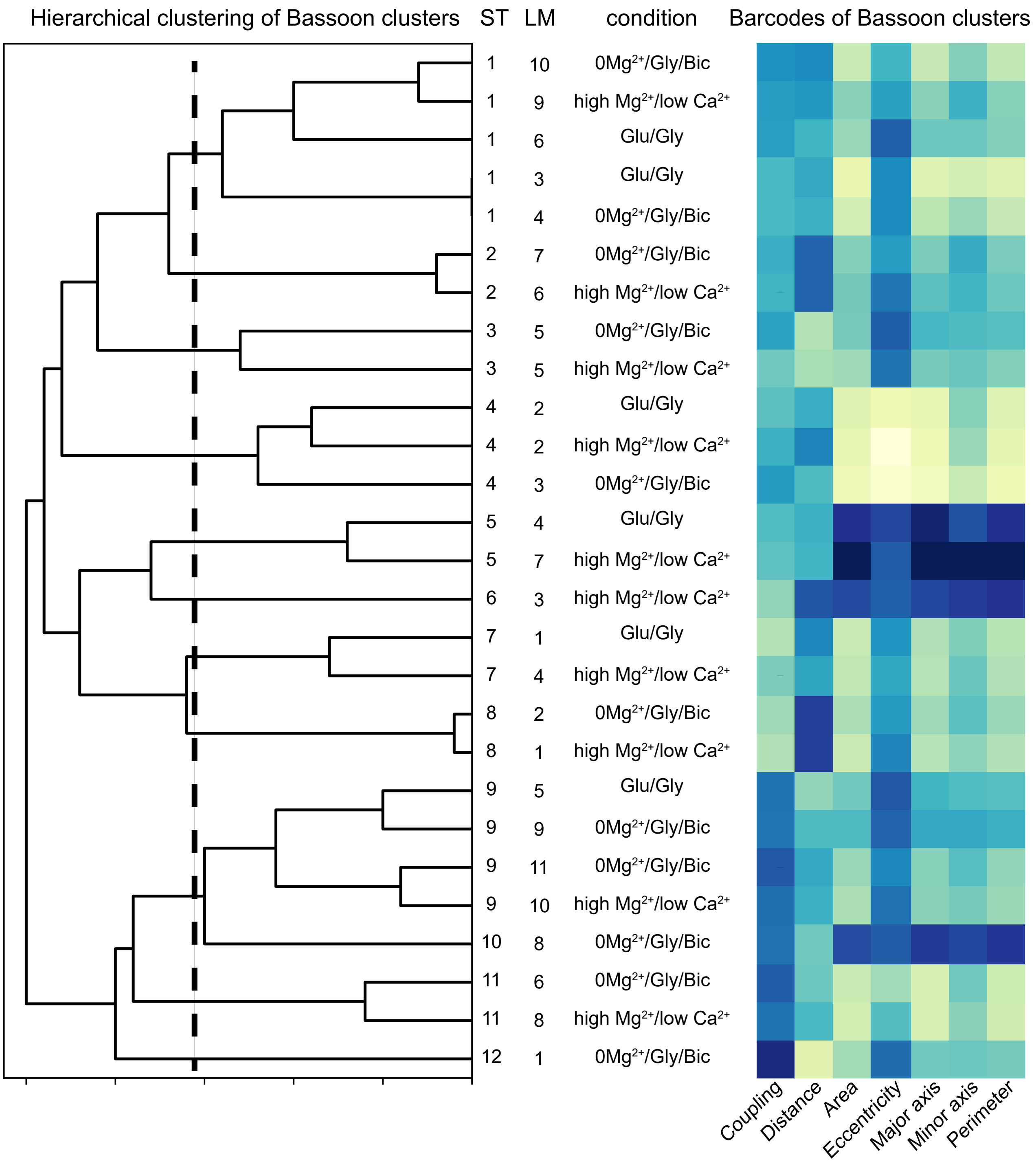

Figure S16: Hierarchical clustering of the main Bassoon subtypes. Subtypes (ST) shown in the dendrogram were determined using the local maxima (LM) identified on the KDE maps of the UMAP embedding for the high Mg^{2+}/low Ca^{2+}, $0Mg^{2+}$/Gly/Bic, and Glu/Gly conditions (Figure 4E,F). The color-code for each feature used in the dendrogram corresponds to the one of Figure S14. The black dotted line identifies the threshold used to select the main synaptic subtypes to be considered for further analysis. The threshold was determined using the silhouette score (see Methods).

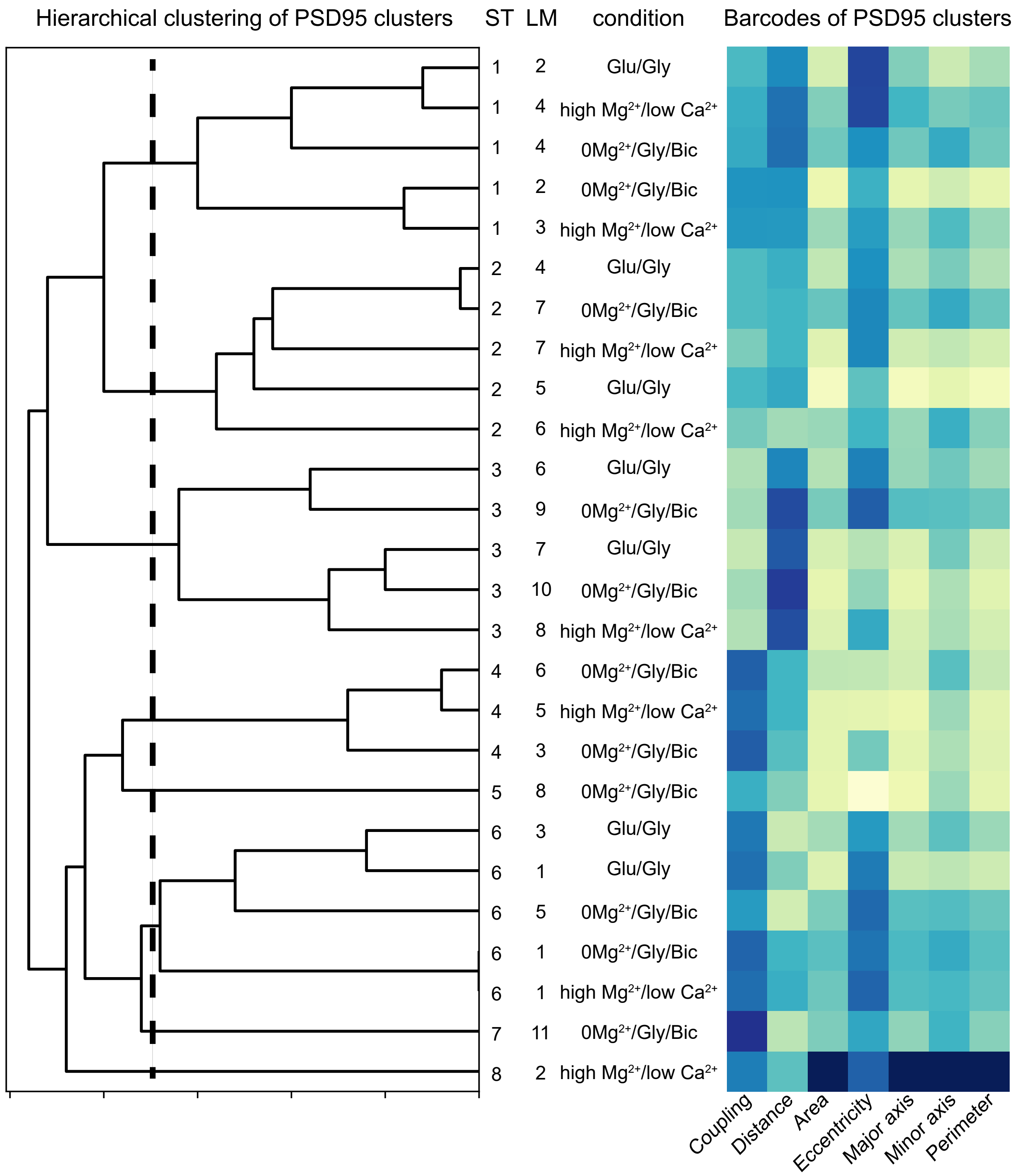

Figure S17: Hierarchical clustering of the main PSD95 subtypes. Subtypes (ST) shown in the dendrogram were determined using the local maxima (LM) identified on the KDE maps of the UMAP embedding for the high Mg^{2+}/low Ca^{2+}, $0Mg^{2+}$/Gly/Bic, and Glu/Gly conditions (Figure S13). The color-code for each feature used in the dendrogram corresponds to the one of Figure S15. The black dotted line identifies the threshold used to select the main synaptic subtypes to be considered for further analysis. The threshold was determined using the silhouette score (see Methods).

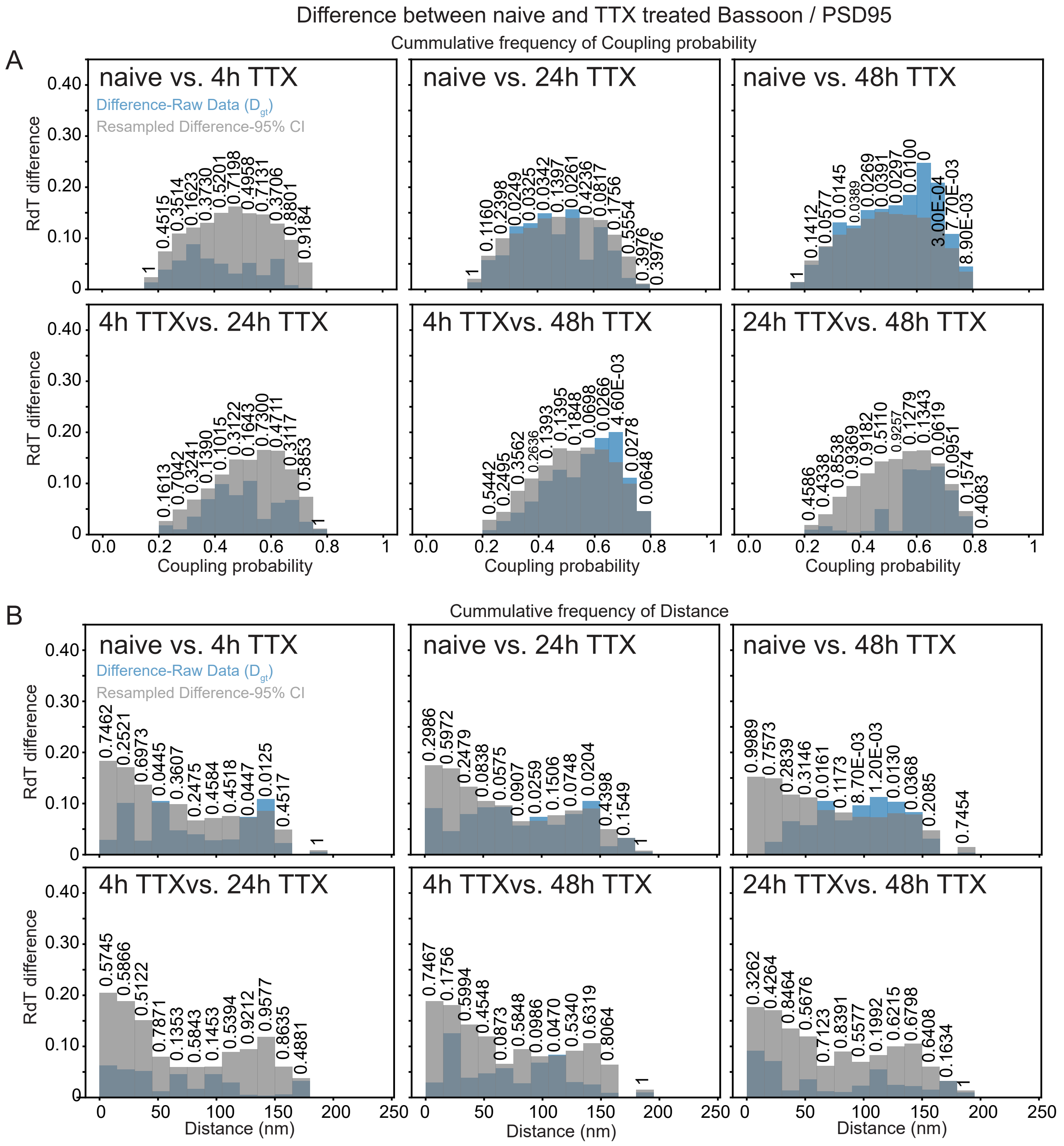

Figure S18: Statistical analysis using a randomization test for the comparison between naive and TTX treated neurons for the protein pair Bassoon and PSD95. The randomization test is performed for the (A) coupling probability and (B) histogram of mean coupling probability at a given distance. The cumulative frequencies of distance is not shown as the resampled F-statistic (see Methods) was not significantly different ($p = 1$). For all panels, the absolute difference between the mean values of raw data (D_{gt}, see Methods) is depicted in blue. The 95% confidence interval of the randomization test difference (D_{rand}, see Methods) is obtained from 10 000 repetitions and shown in grey. We verify the null hypothesis that two conditions belong to the same distribution when the resampled D_{rand} is greater than D_{gt} considering a 95% CI.

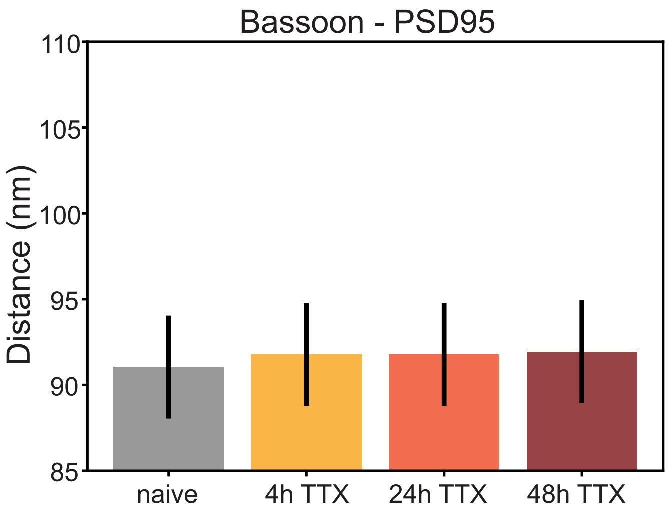

Figure S19: Mean coupling distances calculated for Bassoon and PSD95 cluster pairs for the homeostatic plasticity paradigm (naive - grey, 4h TTX - yellow, 24h TTX - orange, and 48h TTX - red). The error bars correspond to the calculated precision error of 3 nm for the localization of the weighted centroids from the raw STED images (see Methods).

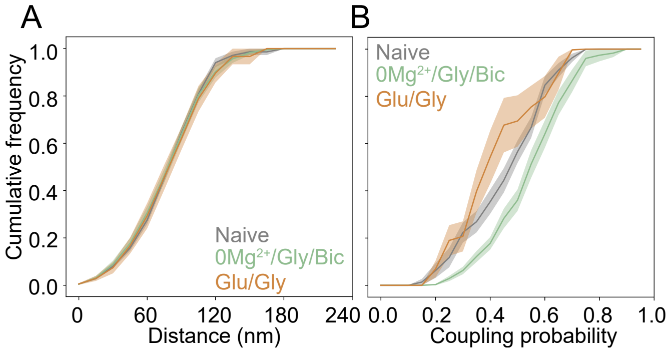

Figure S20: Cumulative frequency curves of the A) coupling distances and B) coupling probability for Bassoon and PSD95 cluster pairs comparing the naive (n = 18, grey) condition with the $0Mg^{2+}$/Gly/Bic (n = 24, green) and Glu/Gly (n = 7, orange) conditions. Mean values for *n* images per condition are shown (solid line) with the standard error of the mean (shaded area).

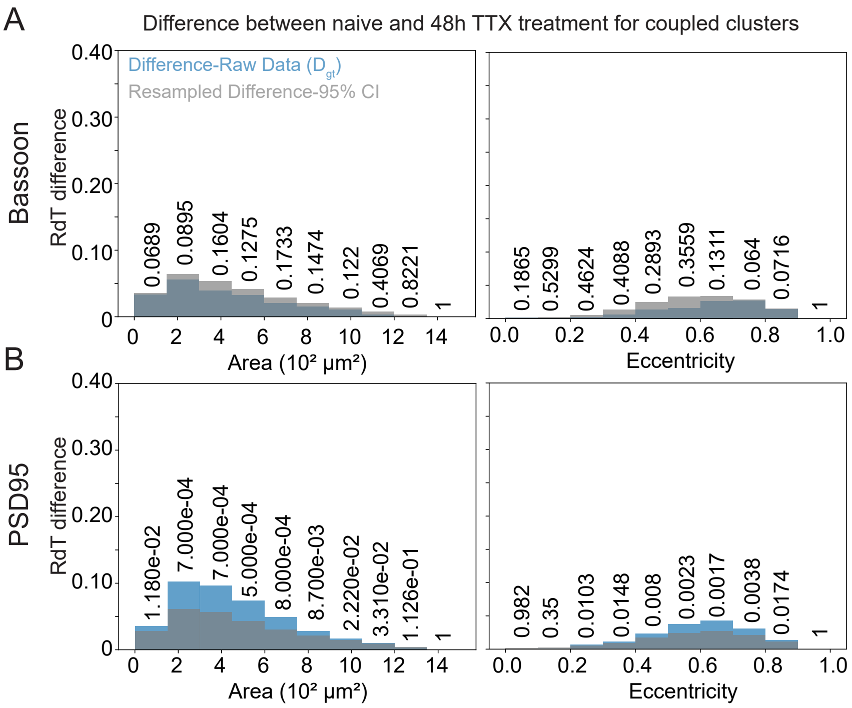

Figure S21: Statistical analysis using a randomization test for the comparison between naive and TTX treated neurons for the cumulative frequencies of the area *(left)*, the eccentricity *(right)* are shown in Figure 6C,D) for coupled clusters of (A) Bassoon and (B) PSD95. For all panels, the absolute difference between the mean values of raw data (D_{gt}, see Methods) is depicted in blue. The 95% confidence interval of the randomization test difference (D_{rand}, see Methods) is obtained from 10 000 repetitions and shown in grey. We verify the null hypothesis that two conditions belong to the same distribution when the resampled D_{rand} is greater than D_{gt} considering a 95% CI.

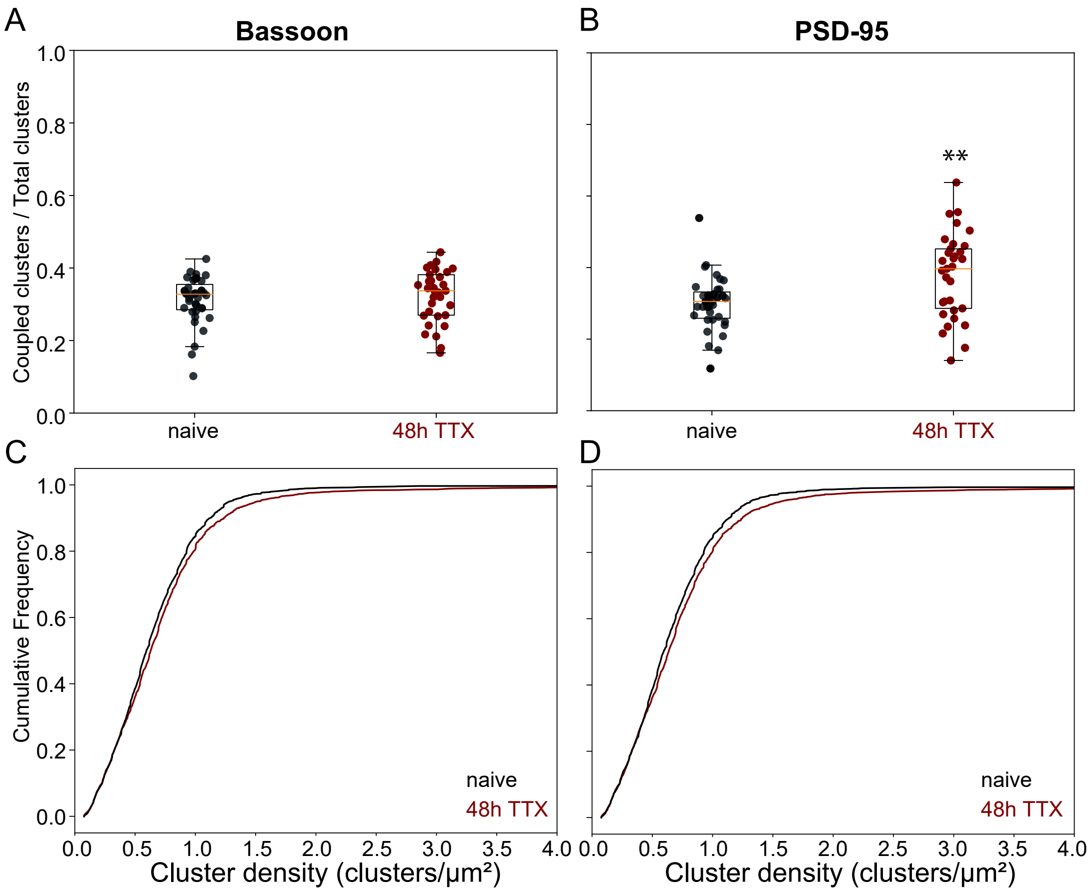

Figure S22: The proportion of coupled PSD95 clusters is increased in the homeostatic plasticity paradigm. Ratios of coupled/total clusters for the homeostatic plasticity experiment showing that the proportion of coupled clusters is not affected by the stimulation for A) Bassoon, while it is significantly increased by a 48h TTX (dark red) treatment for B) PSD95 (t-test: $p = 0.0012$) compared to the naive condition (black). Cumulative frequency curves showing that the density of coupled cluster is significantly increased for C) Bassoon (t-test: $p = 0.0188$) and D) PSD95 (t-test: $p = 0.0043$) by a chronic 48h TTX treatment (dark red - n = 33) compared to the naive condition (black - n = 35) for both proteins.

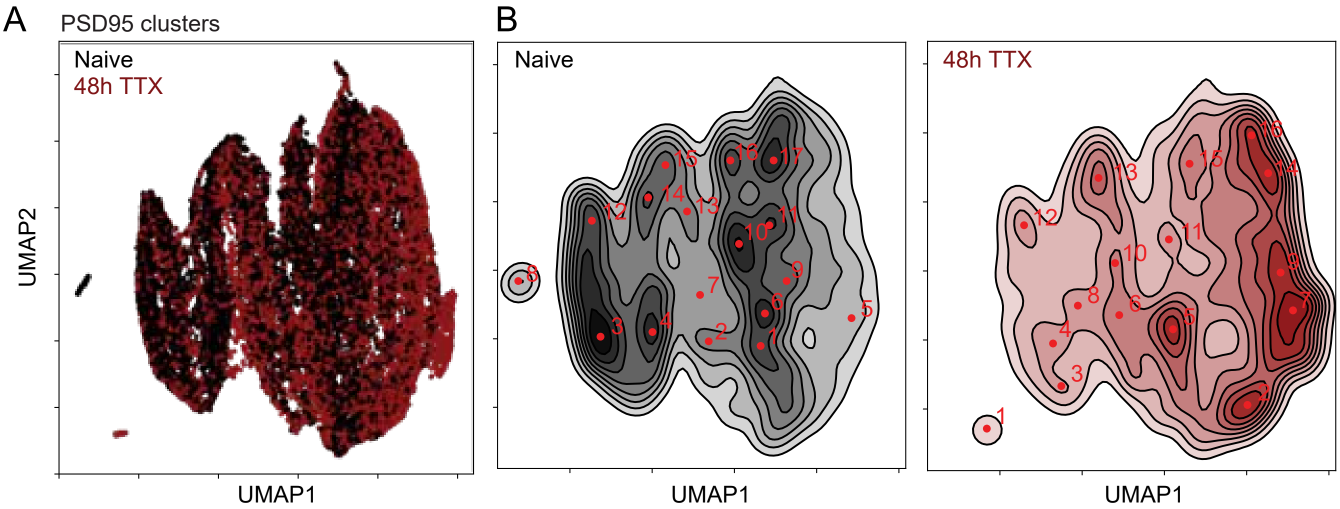

Figure S23: UMAP embedding of the coupling and morphology features for coupled PSD95 clusters in the context of homeostatic plasticity. A) Scatterplot and B) corresponding KDE maps of coupled PSD95 clusters naive (black) or 48h TTX treated cultures (red). The local maxima (numbers in red) identifying synaptic cluster subtypes are indicated on the KDE plots (see Methods).

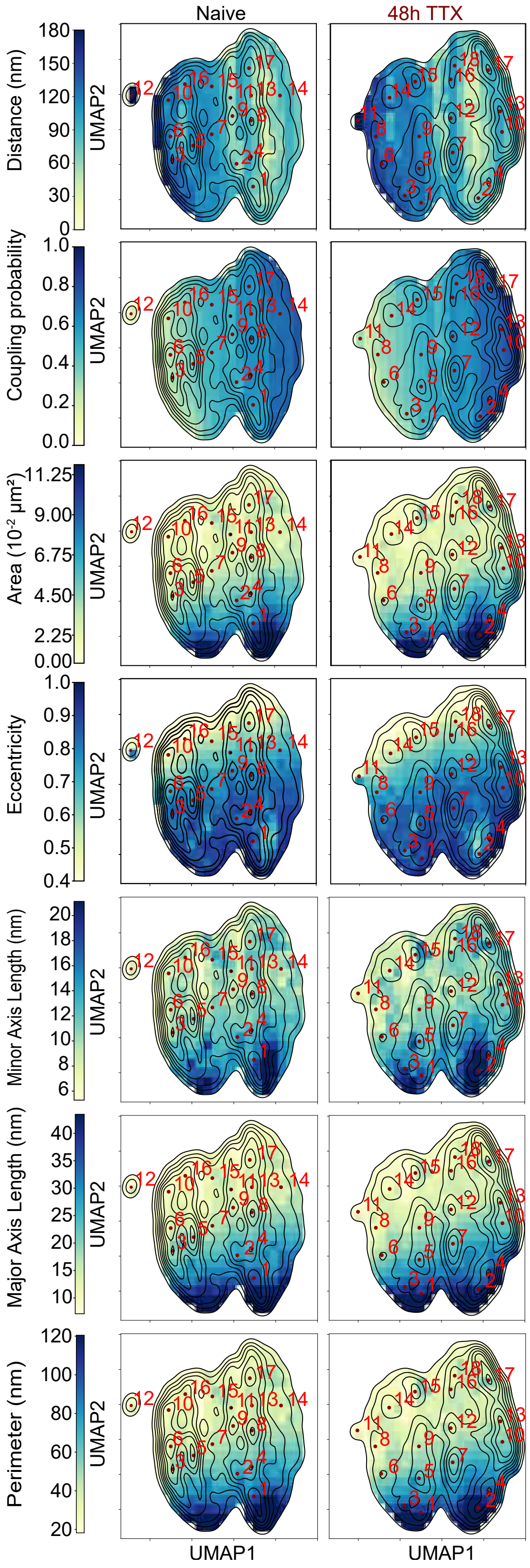

Caption is on the next page

Figure S24: KDE maps of the UMAP embedding overlaid with the colorcoded distribution of coupling and morphological Bassoon cluster features. *(From top to bottom)*: distance, coupling probability, area, eccentricity, minor axis length, major axis length and perimeter. The distributions are compared for the naive *(left)* and 48h TTX treated *(right)* conditions. Minimum (yellow) and maximum (dark blue) values of the color-code are set for each feature: coupling probability (min: 0, max: 1), distance (min: 0 nm, max: 180 nm), area ($0.7 \cdot 10^{-2}$ μm^2, max: $11.8 \cdot 10^{-2}$ μm^2), eccentricity (min: 0.4, max: 1), minor axis length (min: 5.3 nm, max: 21.1 nm), major axis length (min: 7.1 nm, max: 43.5 nm) and perimeter (min: 18.2 nm, max: 120.5 nm).

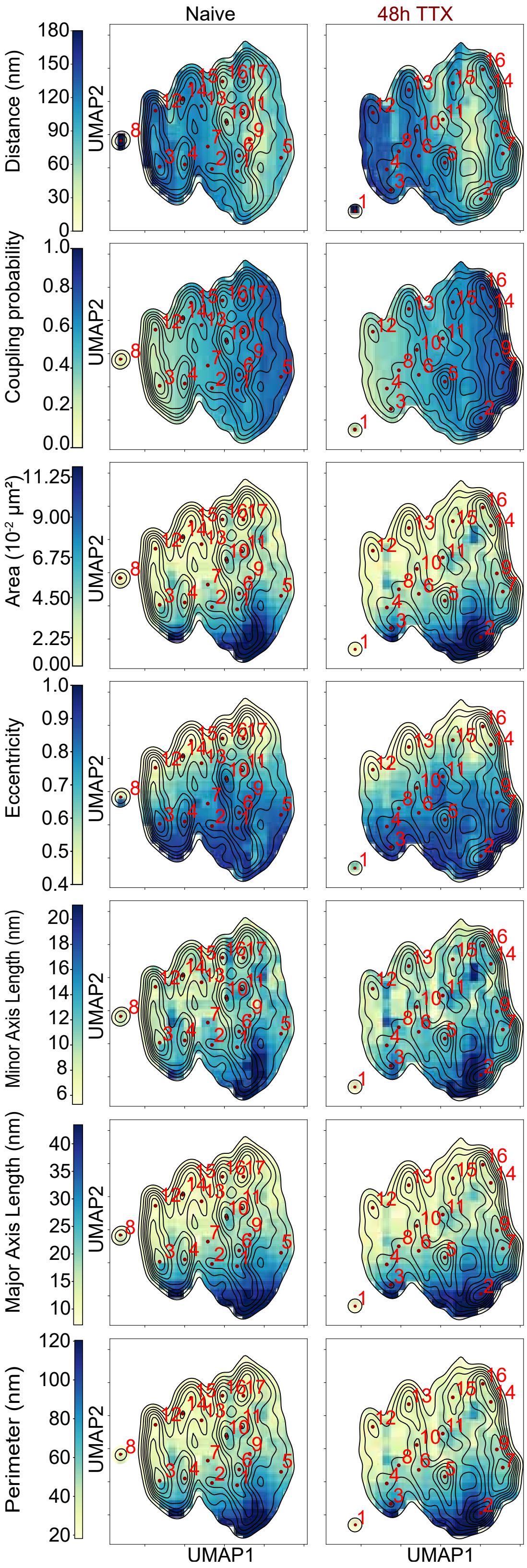

Caption is on the next page

Figure S25: KDE maps of the UMAP embedding overlaid with the color-coded distribution of coupling and morphological PSD95 cluster features. *(From top to bottom)*: distance, coupling probability, area, eccentricity, minor axis length, major axis length and perimeter. The distributions are compared for the naive *(left)* and 48h TTX treated *(right)* conditions. Minimum (yellow) and maximum (dark blue) values of the color-code are set for each feature: coupling probability (min: 0, max: 1), distance (min: 0 nm, max: 180 nm), area ($0.7 \cdot 10^{-2}$ μm^2, max: $11.8 \cdot 10^{-2}$ μm^2), eccentricity (min: 0.4, max: 1), minor axis length (min: 5.3 nm, max: 21.1 nm), major axis length (min: 7.1 nm, max: 43.5 nm) and perimeter (min: 18.2 nm, max: 120.5 nm).

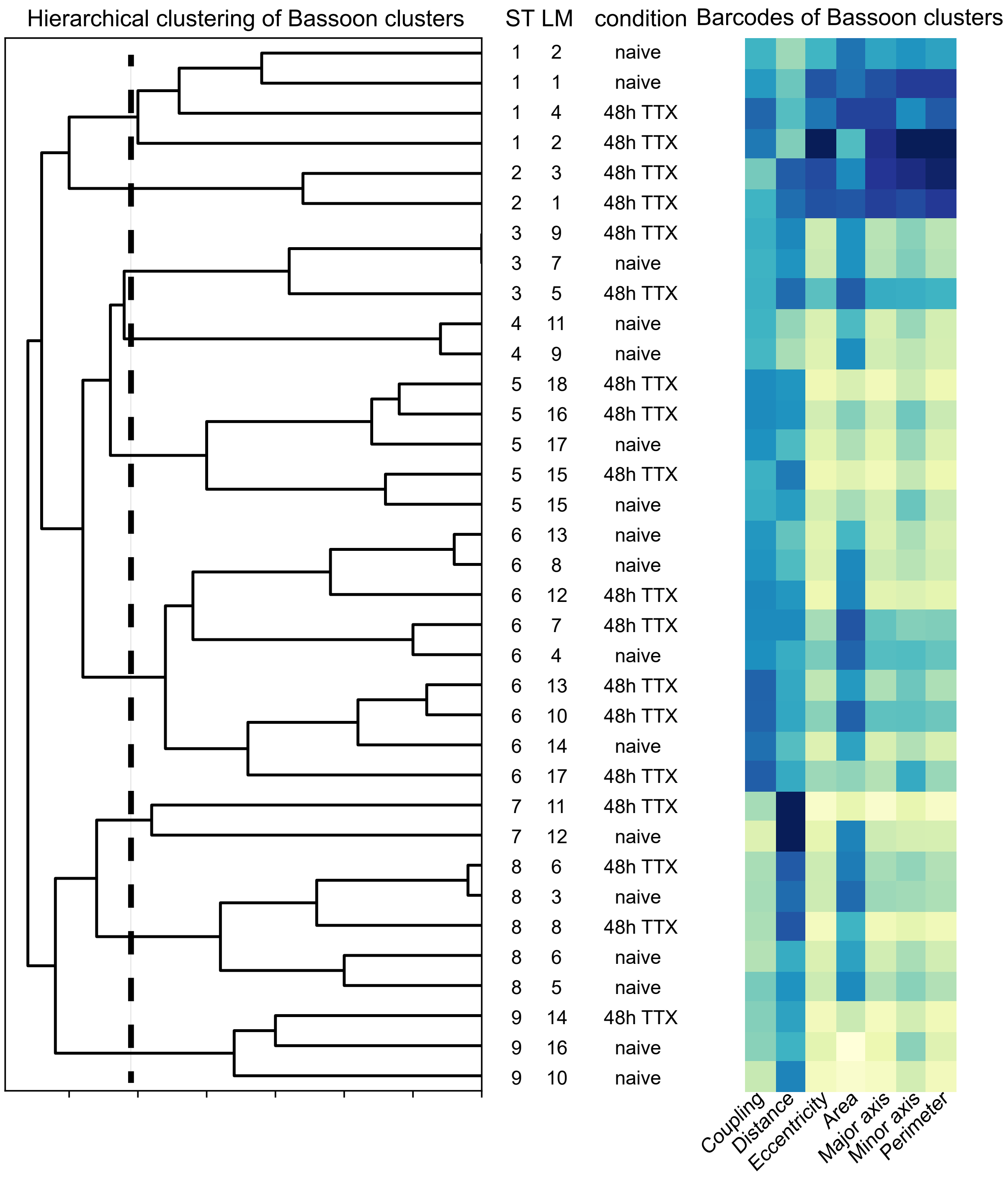

Figure S26: Hierarchical clustering of the main Bassoon synaptic subtypes. Subtypes (ST) shown in the dendrogram were determined using the local maxima (LM) identified on the KDE maps of the UMAP embedding for naive and 48h treated neuronal cultures (Figure 6E,F). The color-code for each feature used in the dendrogram corresponds to the one of Figure S24. The black dotted line identifies the threshold used to identify the main synaptic subtypes to be considered for further analysis. The threshold was determined using the silhouette score (see Methods).

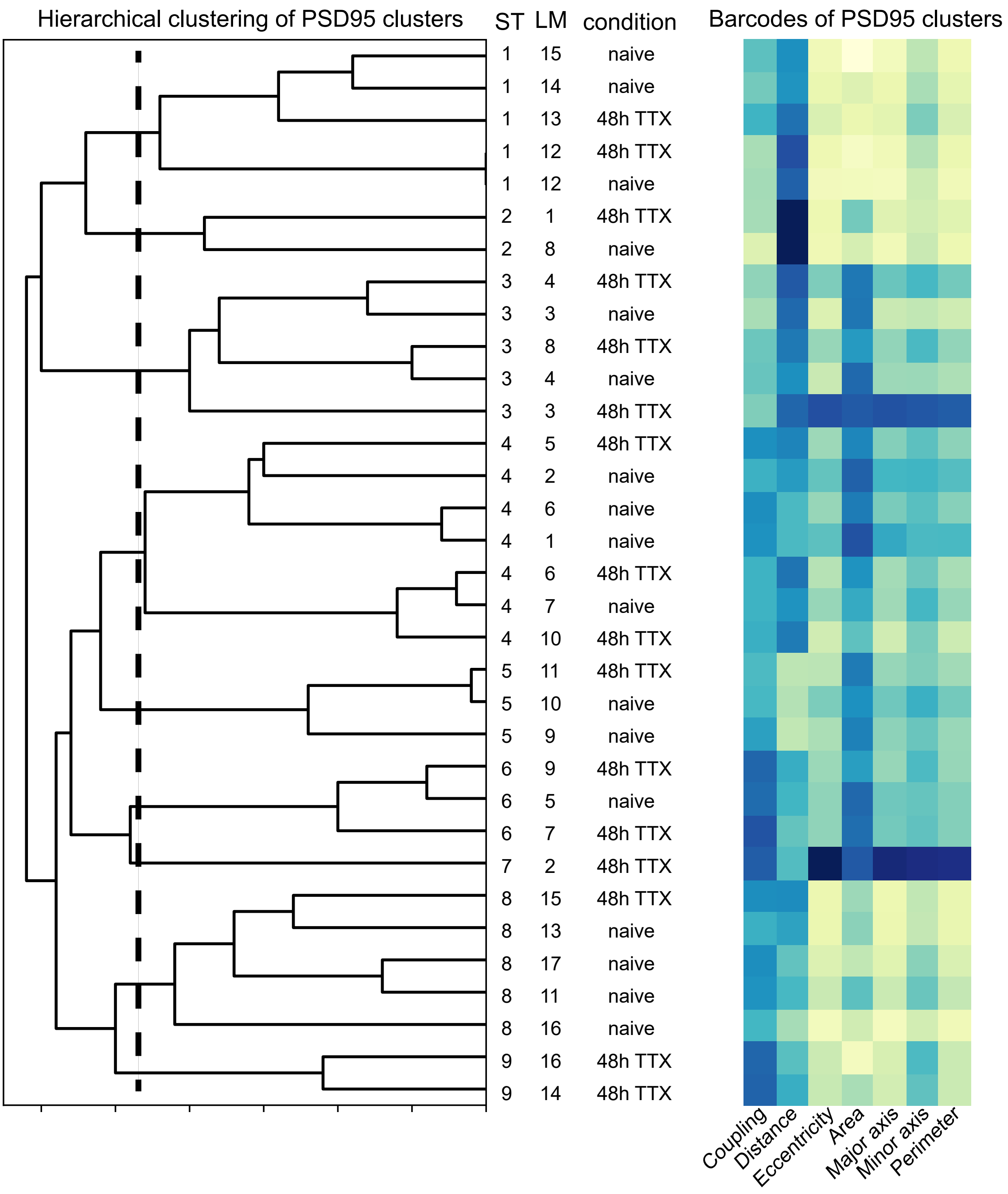

Figure S27: Hierarchical clustering of the main PSD95 synaptic subtypes. Subtypes (ST) shown in the dendrogram were determined using the local maxima (LM) identified on the KDE maps of the UMAP embedding for naive and 48h treated neuronal cultures (Figure S23). The color-code for each feature used in the dendrogram corresponds to the one of Figure S25. The black dotted line identifies the threshold that was selected to identify the main synaptic subtypes for further analysis. The threshold was determined using the silhouette score (see Methods).

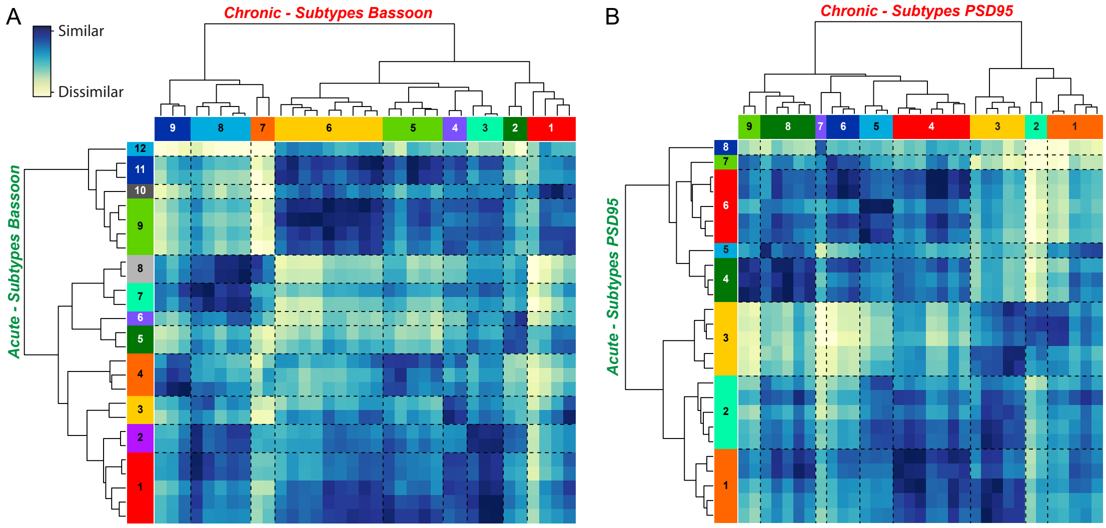

Figure S28: Similarity assessment between the synaptic subtypes identified using hierarchical grouping for the acute and chronic treatment experiments. The Euclidean distance between the groups was measured and used to describe the similarity between the subtypes. The color-code used for the subtypes is in accordance to the one used in Figures 5, 7.

1.2 Tables

Bassoon cluster subtypes	1	2	3	4	5	6	7	8	9	10	11	12
high Mg^{2+}/low Ca^{2+}	13.97%	12.46%	7.12%	11.22%	5.72%	5.04%	3.81%	10.29%	15.99%	5.18%	9.09%	0.10%
$0Mg^{2+}$/Gly/Bic	14.89%	6.71%	6.62%	9.53%	4.41%	3.54%	1.61%	7.06%	21.09%	7.39%	12.73%	4.41%
Glu/Gly	13.18%	2.10%	7.31%	15.42%	9.09%	4.63%	13.90%	8.26%	12.69%	4.35%	8.75%	0.32%
Coupling Probability	0.55	0.51	0.48	0.52	0.45	0.34	0.33	0.31	0.72	0.70	0.72	0.92
Coupling Distance	100.37	132.05	52.74	99.35	91.91	139.21	108.54	150.19	84.38	72.72	79.84	29.38
Area (μm^2)	3.53	4.91	4.63	2.22	11.58	9.66	3.52	3.67	4.84	9.62	3.16	4.15
Eccentricity	0.77	0.79	0.83	0.42	0.87	0.84	0.75	0.77	0.83	0.85	0.63	0.83
Major axis length	17.81	22.04	23.09	11.34	45.05	36.68	17.40	18.11	23.13	38.28	14.50	21.68
Minor axis length	11.10	13.42	12.27	10.22	19.54	18.78	11.52	11.57	12.61	18.16	11.27	11.77
Perimeter	47.34	59.07	60.15	33.46	126.18	108.27	47.12	49.71	60.31	107.43	41.27	58.48

Table S1. Proportion of Bassoon clusters assigned to each main synaptic subtype depending on neuronal activity. The exact values for the coupling and morphological features that were used for the color-coding of the hierarchical grouping are listed for each subtype.

	**high Mg^{2+}/low Ca^{2+} vs. $0Mg^{2+}$/Gly/Bic**	**high Mg^{2+}/low Ca^{2+} vs. Glu/Gly**	**$0Mg^{2+}$/Gly/Bic vs. Glu/Gly**
Chi-square test	1.21×10^{-210}	4.12×10^{-166}	0

Post-hoc chi-square test

**Bassoon cluster subtypes**	**high Mg^{2+}/low Ca^{2+} vs. $0Mg^{2+}$/Gly/Bic**	**high Mg^{2+}/low Ca^{2+} vs. Glu/Gly**	**$0Mg^{2+}$/Gly/Bic vs. Glu/Gly**
1	0.0559	0.2588	0.0123
2	1.46×10^{-48}	1.03×10^{-69}	5.93×10^{-25}
3	0.1492	0.7448	0.1647
4	4.27×10^{-5}	1.25×10^{-10}	6.16×10^{-23}
5	1.06×10^{-5}	8.65×10^{-12}	7.91×10^{-27}
6	4.58×10^{-8}	0.3678	0.0034
7	1.49×10^{-24}	3.72×10^{-95}	9.73×10^{-219}
8	1.41×10^{-17}	5.95×10^{-4}	0.0185
9	7.58×10^{-22}	3.61×10^{-6}	1.55×10^{-28}
10	3.51×10^{-11}	0.0568	2.94×10^{-10}
11	1.52×10^{-17}	0.5747	1.70×10^{-10}
12	6.5×10^{-91}	0.0141	9.00×10^{-31}

Table S2. Results obtained from a Chi-square test to identify significant variation in the proportion of Bassoon synaptic subtypes depending on neuronal activity (see Figure 5).

**PSD95 cluster subtypes**	1	2	3	4	5	6	7	8
high Mg^{2+}/low Ca^{2+}	24.67%	14.13%	19.79%	9.93%	4.11%	23.95%	0.00%	3.42%
$0Mg^{2+}$/Gly/Bic	18.36%	9.16%	12.70%	13.43%	4.06%	31.67%	5.56%	5.05%
Glu/Gly	7.19%	28.38%	24.15%	7.04%	6.84%	24.27%	0.00%	2.13%
Coupling Probability	0.55	0.44	0.29	0.73	0.52	0.68	0.89	0.67
Coupling Distance	117.34	85.56	138.99	87.26	66.71	67.13	48.62	80.11
Area (μm^2)	3.85	3.47	3.35	2.85	2.29	4.53	4.86	17.59
Eccentricity	0.80	0.74	0.71	0.56	0.41	0.81	0.73	0.84
Major axis length	19.88	17.07	16.81	13.18	11.48	21.37	19.71	53.92
Minor axis length	11.15	11.14	11.13	10.89	10.43	12.23	13.25	27.02
Perimeter	51.07	46.02	45.33	38.22	34.03	56.16	55.33	173.71

Table S3. Proportion of PSD95 clusters assigned to each main synaptic subtype depending on neuronal activity. The exact values for the coupling and morphological features that were used for the color-coding of the hierarchical grouping are listed for each subtype.

	**high Mg^{2+}/low Ca^{2+} vs. $0Mg^{2+}$/Gly/Bic**	**high Mg^{2+}/low Ca^{2+} vs. Glu/Gly**	**$0Mg^{2+}$/Gly/Bic vs. Glu/Gly**
Chi-square test	1.33×10^{-240}	2.22×10^{-164}	0
	**Post-hoc chi-square test**		
**PSD95 cluster subtypes**	**high Mg^{2+}/low Ca^{2+} vs. $0Mg^{2+}$/Gly/Bic**	**high Mg^{2+}/low Ca^{2+} vs. Glu/Gly**	**$0Mg^{2+}$/Gly/Bic vs. Glu/Gly**
1	5.77×10^{-30}	6.72×10^{-108}	6.63×10^{-57}
2	9.36×10^{-31}	1.09×10^{-78}	7.93×10^{-190}
3	2.16×10^{-46}	6.86×10^{-08}	1.03×10^{-61}
4	1.80×10^{-15}	4.92×10^{-7}	1.89×10^{-24}
5	0.8658	1.69×10^{-10}	8.79×10^{-12}
6	1.26×10^{-36}	0.7248	4.32×10^{-17}
7	4.64×10^{-122}	0.9641	4.32×10^{-45}
8	4.48×10^{-9}	1.85×10^{-4}	1.60×10^{-13}

Table S4. Results obtained from a Chi-square test to identify significant variation in the proportion of PSD95 synaptic subtypes depending on neuronal activity (see Figure 5).

**Bassoon cluster subtypes**	1	2	3	4	5	6	7	8	9
naive	14.43%	5.23%	6.52%	7.46%	12.86%	23.14%	1.30%	20.07%	8.98%
48h TTX	16.65%	5.01%	9.22%	4.07%	13.63%	38.43%	1.06%	7.92%	4.00%
Coupling Probability	0.63	0.45	0.51	0.50	0.59	0.67	0.24	0.32	0.32
Coupling Distance	70.50	131.58	118.32	57.94	106.64	95.22	186.50	122.12	103.66
Area (μm^2)	9.54	9.50	4.08	2.70	2.48	3.49	1.77	2.81	1.89
Eccentricity	0.80	0.82	0.80	0.73	0.56	0.77	0.64	0.77	0.44
Major axis length	34.96	38.27	20.38	14.68	12.73	17.93	12.04	16.09	10.48
Minor axis length	19.31	18.85	11.90	10.00	10.50	11.01	8.02	9.92	9.37
Perimeter	107.78	111.61	54.24	39.55	36.57	47.61	31.48	42.45	30.64

Table S5. Proportion of Bassoon clusters assigned to each main synaptic subtype in the context of homeostatic plasticity. The exact values for the coupling and morphological features that were used for the color-coding of the hierarchical grouping are listed for each subtype.

	**naive vs. 48h TTX**
Chi-square test	0
**Post-hoc chi-square test**	
**Basson cluster subtypes**	**naive vs. 48h TTX**
1	4.39×10^{-8}
2	0.3374
3	3.50×10^{-19}
4	3.17×10^{-38}
5	0.0444
6	1.22×10^{-193}
7	0.0536
8	3.86×10^{-213}
9	1.99×10^{-72}

Table S6. Results obtained from a Chi-square test to identify significant variation in the proportion of PSD95 synaptic subtypes for the homeostatic activity paradigm (see Figure 7).

PSD95 cluster subtypes	1	2	3	4	5	6	7	8	9
naive	15.33%	1.25%	20.41%	18.60%	9.91%	5.32%	3.77%	20.47%	4.95%
48h TTX	9.17%	0.94%	10.87%	19.27%	7.19%	19.85%	8.21%	12.90%	11.61%
Coupling Probability	0.39	0.24	0.37	0.56	0.51	0.74	0.75	0.58	0.73
Coupling Distance	124.84	187.83	126.77	107.72	48.47	87.25	83.09	87.17	87.93
Area (μm^2)	2.10	1.99	4.96	4.43	4.21	4.46	12.56	2.39	3.39
Eccentricity	0.45	0.58	0.81	0.78	0.79	0.80	0.86	0.59	0.52
Major axis length	11.14	12.10	22.44	20.80	20.30	20.92	40.98	12.51	14.56
Minor axis length	9.91	8.94	12.62	12.41	12.16	12.32	19.69	10.03	12.45
Perimeter	32.46	33.02	59.32	55.01	53.79	54.38	110.70	35.16	42.78

Table S7. Proportion of PSD95 clusters assigned to each main synaptic subtype in the context of homeostatic plasticity. The exact values for the coupling and morphological features that were used for the color-coding of the hierarchical grouping are listed for each subtype.

	**naive vs. 48h TTX**
Chi-square test	0
**Post-hoc Chi-square test**	
**PSD95 cluster subtypes**	**naive vs. 48h TTX**
1	5.01×10^{-63}
2	0.0089
3	2.73×10^{-121}
4	0.1281
5	3.82×10^{-18}
6	0
7	2.43×10^{-63}
8	2.18×10^{-73}
9	7.29×10^{-105}

Table S8. Results obtained from a Chi-square test to identify significant variation in the proportion of Bassoon synaptic subtypes for the homeostatic activity paradigm (see Figure 7).

REFERENCES

Ta, H., Keller, J., Haltmeier, M., Saka, S. K., Schmied, J., Opazo, F., et al. (2015). Mapping molecules in scanning far-field fluorescence nanoscopy. *Nature communications* 6, 1–7
